# Treatment-Resistant to Antipsychotics: A Resistance to Everything? Psychotherapy in Treatment-Resistant Schizophrenia and Nonaffective Psychosis: A 25-Year Systematic Review and Exploratory Meta-Analysis

**DOI:** 10.3389/fpsyt.2019.00210

**Published:** 2019-04-17

**Authors:** Daniela Polese, Michele Fornaro, Mario Palermo, Vincenzo De Luca, Andrea de Bartolomeis

**Affiliations:** ^1^Treatment Resistant Psychosis Unit and Laboratory of Molecular and Translational Psychiatry, Section of Psychiatry, University School of Medicine of Naples “Federico II”, Naples, Italy; ^2^Department of Neuroscience, Psychiatric Unit, Sant’Andrea University Hospital, “Sapienza” University of Rome, Rome, Italy; ^3^Centre for Addiction and Mental Health, Toronto, Canada; ^4^Department of Psychiatry, University of Toronto, Toronto, ON, Canada

**Keywords:** treatment-resistant psychosis, dopamine supersensitivity, negative symptoms, psychotherapy, behavioral therapy, group psychotherapy, positive symptoms

## Abstract

**Background:** Roughly 30% of schizophrenia patients fail to respond to at least two antipsychotic trials. Psychosis has been traditionally considered to be poorly sensitive to psychotherapy. Nevertheless, there is increasing evidence that psychological interventions could be considered in treatment-resistant psychosis (TRP). Despite the relevance of the issue and the emerging neurobiological underpinnings, no systematic reviews have been published. Here, we show a systematic review of psychotherapy interventions in TRP patients of the last 25 years.

**Methods:** The MEDLINE/PubMed, ISI WEB of Knowledge, and Scopus databases were inquired from January 1, 1993, to August 1, 2018, for reports documenting augmentation or substitution with psychotherapy for treatment-resistant schizophrenia (TRS) and TRP patients. Quantitative data fetched by Randomized Controlled Trials (RCTs) were pooled for explorative meta-analysis.

**Results:** Forty-two articles have been found. Cognitive behavioral therapy (CBT) was the most frequently recommended psychotherapy intervention for TRS (studies, *n* = 32, 76.2%), showing efficacy for general psychopathology and positive symptoms as documented by most of the studies, but with uncertain efficacy on negative symptoms. Other interventions showed similar results. The usefulness of group therapy was supported by the obtained evidence. Few studies focused on negative symptoms. Promising results were also reported for resistant early psychosis.

**Limitations:** Measurement and publication bias due to the intrinsic limitations of the appraised original studies.

**Conclusions:** CBT, psychosocial intervention, supportive counseling, psychodynamic psychotherapy, and other psychological interventions can be recommended for clinical practice. More studies are needed, especially for non-CBT interventions and for all psychotherapies on negative symptoms.

## Introduction

Schizophrenia affects approximately 1% of the population, usually starting in adolescence or young adulthood, frequently leading to persistent disability, with a high risk of suicide (8%). Despite the advance in antipsychotics treatment, approximately 30% of patients with schizophrenia show a poor response or no response to antipsychotics (1–7), demonstrating persistent positive symptoms (i.e., hallucinations, delusions). The experience of persistent delusions and hallucinations may result in further disability, poor prognosis, and risk of suicide (8, 9). Finally, treatment-resistant psychosis (TRP) is responsible for increasing health assistance expenditure. For instance, in the United States, treatment-resistant schizophrenia (TRS) adds more than 34 billion dollars in the annual direct medical costs (10).

In the presence of pharmacological treatment resistance, can nonpharmacological, psychotherapy-based interventions significantly overcome the therapeutic response deadlock? Which psychotherapy in combination with antipsychotics does work better? Finally, what are the limitations and the pitfalls of the research on psychotherapy in TRS and TRP?

This review aims to provide a critical, systematic overview covering the last 25 years of published results of all types of psychotherapy, as adjunctive or substitutive therapy, specifically in TRS or TRP patients, including early psychosis and psychotic onset. TRS and TRP for many patients are lifelong mental disorders with significant consequences on most functional domains (11, 12). TRS represents a severe condition with relevant clinical, social, and health costs and consequences (2). In clinical practice, the criteria to define TRS have not been always consistent over time (2). The first complete definition was introduced in the seminal article of Kane and collaborators (13) on clozapine efficacy in TRS. Most of the new proposed criteria require the lack of response to at least two consecutive treatments with antipsychotics; in most cases, one of the two antipsychotics should be an atypical one, of adequate dose and duration (≥6 weeks). An adequate dose of antipsychotic medication in the most recent report is defined as a daily dose of ≥400 mg chlorpromazine equivalence (14–17). The lack of response has been indicated as a relative change in the evaluation scales (i.e., ≥20% decrease in the Positive and Negative Syndrome Scale) (17). Psychotic symptom persistence has been demonstrated to cause distress and serious interference with functioning (18), complicating the clinical course of schizophrenia. Therefore, a large proportion of patients may never reach a functional recovery (19). These patients show poor global functioning and life quality (20, 21), increased drug abuse (6), and reduced cognitive performance compared to patients who respond to the treatment (22). Persistent psychotic symptoms have been observed for 2 years after the initiation of symptoms in 15% of cases (23). In a 15-year follow-up study of patients affected by nonaffective psychosis, every psychotic episode has resulted in raising the probability to experience residual positive symptoms. At least 25% of patients showed persistent positive and negative symptoms after the first episode, while nearly 50% presented persistent symptoms after the fourth episode (24). According to this progression of symptoms persistence, the total number of treatment-resistant patients can increase up to 60% (25). Two forms of treatment resistance have been hypothesized: a type of resistance that is already present at the onset of the pathology, and a second one that develops later on during the trajectory of the disorder and after a period of successful response to antipsychotics (26–28). Remarkably, 82% of TRS had been reported to be resistant since their first episode of psychosis, while 18% of patients with TRS develop resistance after a period of adequate response. It has been reported that the first group could recognize a neurodevelopmental disorder with relatively normal dopaminergic function and prevalent aberrant cortical–subcortical dysfunction (29, 30). Clozapine, the prototypical second-generation antipsychotic, is considered the gold standard of pharmacological treatment for TRS (31–34), even if its superiority in comparison to other second-generation antipsychotics has been challenged in recent meta-analysis (16, 35, 36). Moreover, drug combinations strategies are often used in TRP (32, 37–39) and in the “ultraresistant patients,” who do not respond or respond only partially to clozapine. It has been estimated that approximately 30% of patients who are treated with clozapine do not respond adequately (14, 40, 41). Clinical features at diagnosis can only partially predict resistance to the treatment: poorer premorbid functions, an earlier age at onset of positive symptoms, family history of schizophrenia spectrum disorder, longer duration of untreated psychosis (DUP) (26, 42–48), male gender, a history of specific substance abuse, severe negative symptoms, and presence of soft neurological signs (3, 23, 42, 47, 49–51). Functional and structural brain imaging has identified potential brain abnormalities related to treatment response or resistance, specifically at the level of the frontal cortex, basal ganglia, corpus callosum, and anterior cingulate. Nevertheless, correlations with brain abnormalities have still not been consistently replicated (52, 53). In our study, we included an exploratory meta-analysis to provide a quantitative synthesis of data from Randomized Controlled Trials (RCTs). The aim of this latter analysis was to compare the efficacy of an augmentation approach with cognitive behavioral therapy (CBT) versus treatment as usual (TAU) in patients with treatment-resistant schizophrenia.

### Psychotherapy Approach to Psychosis

The so-called “Dodo Bird Verdict” has been suggested in many reports to indicate that different psychological therapies are of nonspecific or similar efficacy, but this view is controversial and can be contrasted by meta-analytic studies (54–59). Criteria to define evidence-based psychotherapy (EBP) have been established in youth psychotherapy (60). The comparison between EBP and the usual care has shown a more effective performance in the former but advantages in the latter (61, 62). Some researchers have used befriending (BF), an atheoretical and manualized control therapy (63), as a nonspecific relationship that works as a control group, but it has been shown that this approach could have a therapeutic impact, too (64). Nevertheless, psychological interventions have become more widely accepted over the past two decades (65–67). The majority of recent publications consider CBT the elective psychotherapy for psychosis (68, 70) and other treatments are not frequently studied. In particular, the number of articles on the psychodynamic treatment of schizophrenia was very high from 1966 to 1987, with the decline starting after 1980; however, no one was centered on treatment-resistant schizophrenia (71). Mueser et al. observed that the published studies are “only a crude index of the current therapy in schizophrenia since a small fraction of psychodynamic psychotherapy practitioners publishes their treatment cases.” In the history of psychodynamic psychiatry and psychoanalysis, psychosis has been traditionally considered impervious to treatment. However, recent literature points out to the association between environmental factors, such as childhood adversity, and the development of psychotic experiences, psychotic symptoms, and diseases (72–79). In fact, trajectory-based approaches to study clinical consequences to potentially traumatic events (PTEs) have recently emerged. In particular, prototypical trajectories have been found across independent studies, and resilience seems to determine the modal response to adversity (80). Abnormal early-life experience, such as early relationships characterized by a “lack of affectivity” during the first year of life, has been suggested to be potentially pathogenic (81). This aspect should also be evaluated as psychologically determinant in contributing to the development of a psychotic disorder. Furthermore, recent literature has also shown the important role played by the therapeutic relationships in all psychiatric settings in predicting the outcome (82–84). It has also been evidenced how therapist attitude and characteristics in the relationship can influence the outcome specifically in TRS patients (85).

Therefore, in the last 20 years, there has been a growing interest in developing a psychological intervention for people who continue to experience psychotic symptoms despite adequate pharmacological treatment (14, 86–90). In early interventions on psychosis, psychotherapy is a potentially relevant part of the treatment, whereas the medication only might neither be sufficient nor efficient (44, 91–96). Medications can also determine a worse clinical condition and be detrimental, since they can have brain structural effects (97–99). Remarkably, antipsychotic treatment can result in further psychotic symptomatology at this stage, due to a dopaminergic supersensitivity effect, induced by the treatment itself (100–102). It has been observed that early psychosis patients may present treatment resistance. In particular, approximately 20% continue to have significant residual positive symptoms after 12 weeks of comprehensive treatment (103). Nevertheless, in early psychosis, a psychological or an integrated therapy with an adequate dose of medication could be effective, maximize results, prevent relapses, achieve recovery, and overcome drug resistance. Studies on the prodromal phase of psychosis have demonstrated that psychological treatments can be effective in reducing transition to psychosis (103, 104). Also, studies on psychosis onset have shown that, in selected cases, psychological interventions can be more appropriated as the first choice than medications (86, 105–107). The National Institute for Clinical Excellence (NICE) (108) and the Schizophrenia Patient Outcome Report Team (PORT) guidance included cognitive behavioral therapy (CBT) in their preferred list of treatments for schizophrenia (108, 109).

## Materials and Methods

**Figure 1:**
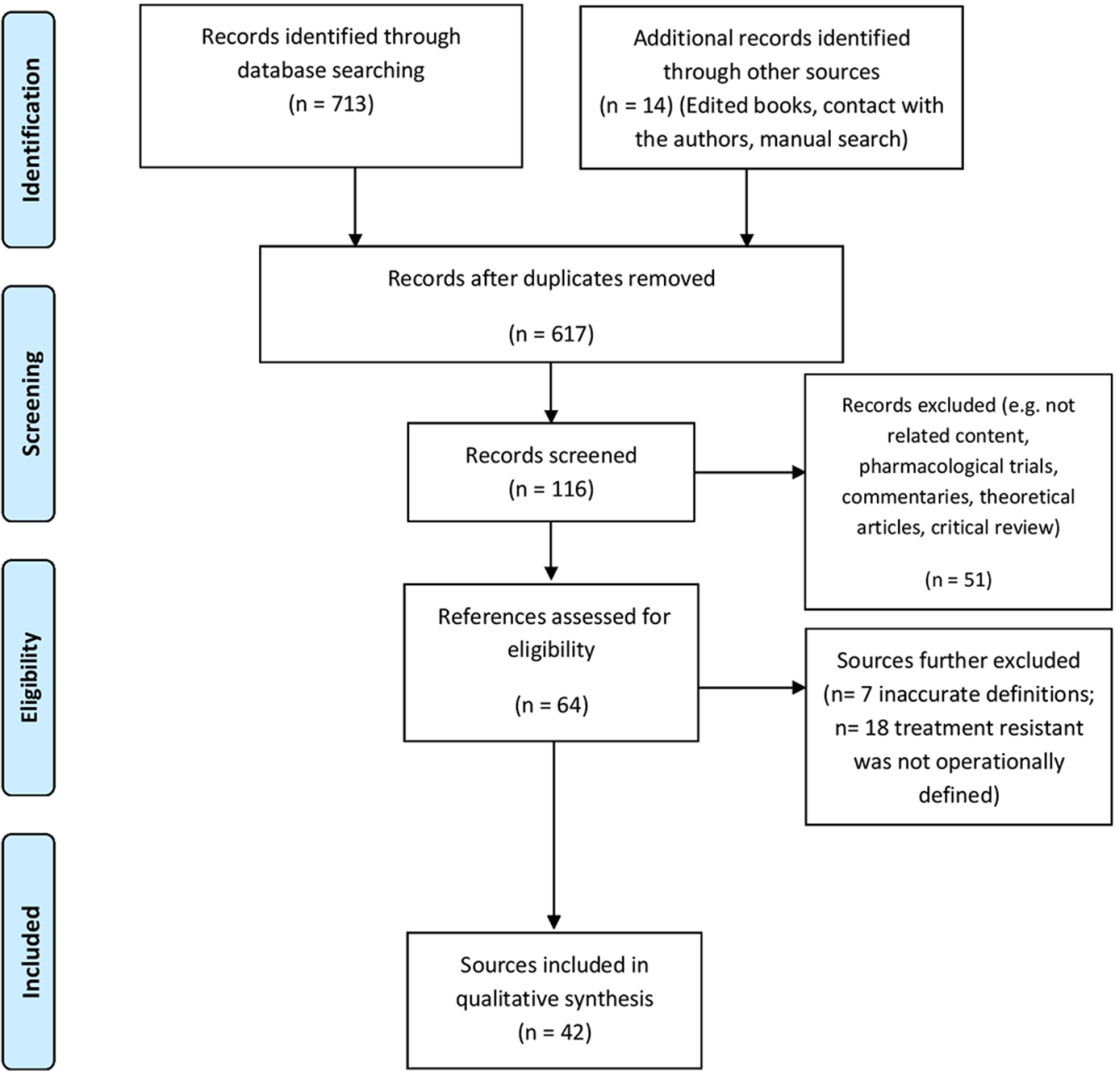
Flow chart of review procedures.

Aimed at achieving a high standard of reporting, we followed the procedures indicated by the 2009 update of the Preferred Reporting Items for Systematic reviews and Meta-Analyses (PRISMA) guidelines (http://www.prisma-statement.org/) (see **Figure 1**) (110).

### Eligibility Criteria, Information Sources, and Search Strategy

We limited our search to those records related to TRP, TRS and psychotherapy of the last 25 years, from January 1, 1993, until August 1, 2018. Such timeframe owed to methodological considerations aimed at including studies relying on homogeneous diagnostic criteria. A systematic database search was performed on MEDLINE/PubMed, Web of Science/ISI Web of Knowledge, and Scopus. The following combinations of keywords have been used: “treatment resistant psychosis OR treatment resistance psychosis AND treatment-resistant schizophrenia OR treatment resistance schizophrenia AND psychotherapy,” “antipsychotic resistant response OR antipsychotic resistance response AND psychotherapy,” “clozapine resistance AND psychotherapy OR augmentation strategies,” “partial responders antipsychotics AND psychotherapy OR augmentation psychotherapy,” “clozapine non responders AND/OR poor responder antipsychotics AND psychotherapy OR augmentation psychotherapy,” “psychosis AND antipsychotics psychotherapy augmentation,” “medical resistance AND psychosis psychotherapy,” “treatment resistant OR treatment-resistant OR treatment resistance OR treatment-resistance AND psychosis AND/OR schizophrenia AND psychotherapy AND/OR psychodynamic psychotherapy AND/OR therapeutic relationship.” RCT, meta-analyses relevant open-label trials, significant articles, including case reports, controlled and uncontrolled trials, and ongoing trials of pharmacological treatments, augmented or substituted with psychotherapeutic approaches to TRP and TRS, have been selected. No language restriction was applied, and relevant cross-references were retrieved as necessary. Studies concerning augmentation or substitution with medication have been excluded. Articles referring to TR in different pathologies from nonaffective psychosis and schizophrenia spectrum disorders have also been excluded. To overcome the problem of nonspecificity in psychotherapy, particular attention has been paid to the psychotherapy method and its details and to the control groups. Critical and systematic reviews on psychological interventions in TRP and TRS have been considered for a further review of literature. The most frequent cluster of symptoms measured by clinical scale assessments that have been included are 1) general psychopathology, 2) positive and negative symptoms, 3) cognitive symptoms, 4) affective symptoms, and 5) social functioning. The following aspects have been considered: 1) the stage of illness, such as the prodromal phase, the onset, any time after the onset and during the chronic phase; 2) the population of patients regarding diagnosis, duration of illness, age, age of onset, and duration of untreated psychosis (DUP); and 3) the type of psychotherapy, such as individual or group, duration of the treatment, frequency and time of the sessions, type of comparison or control group (if present), and blindness of the raters.

**Figure 2:**
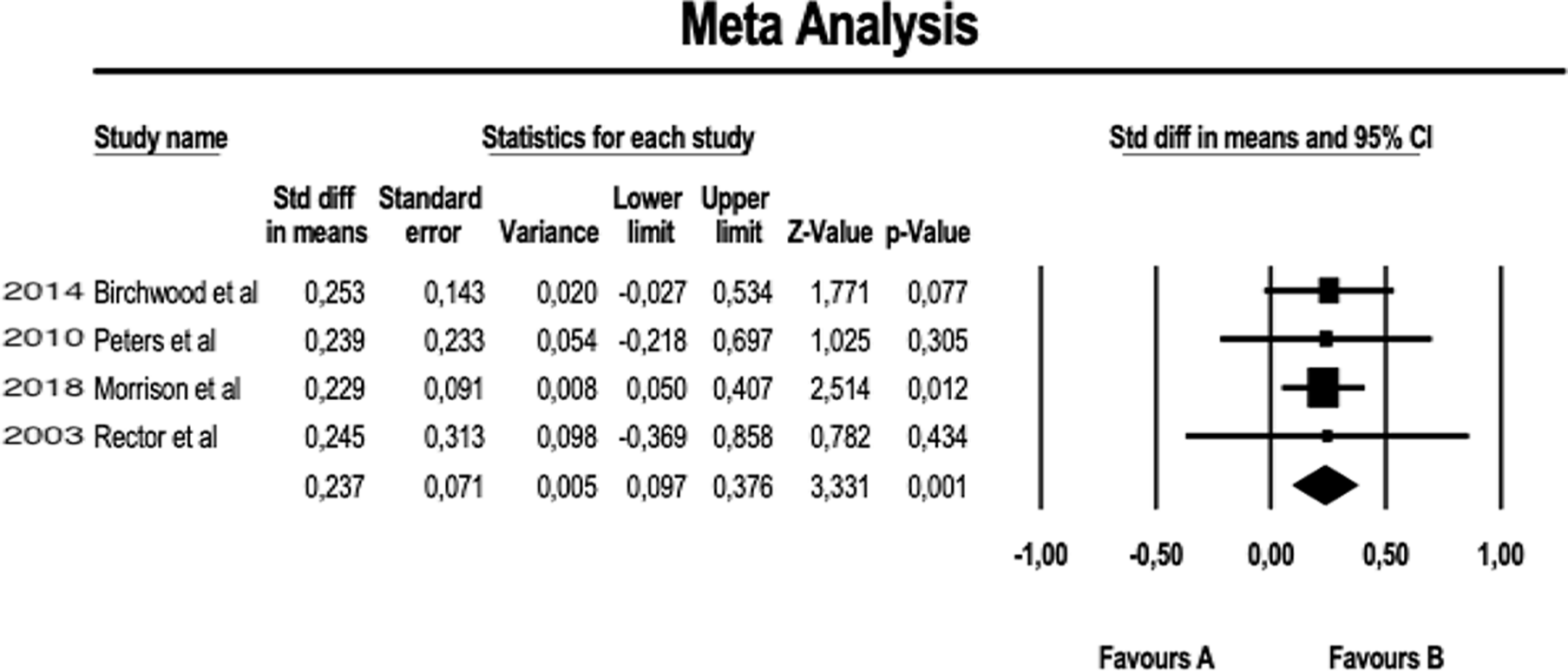
Meta-analysis of PANSS positive symptoms.

**Figure 3:**
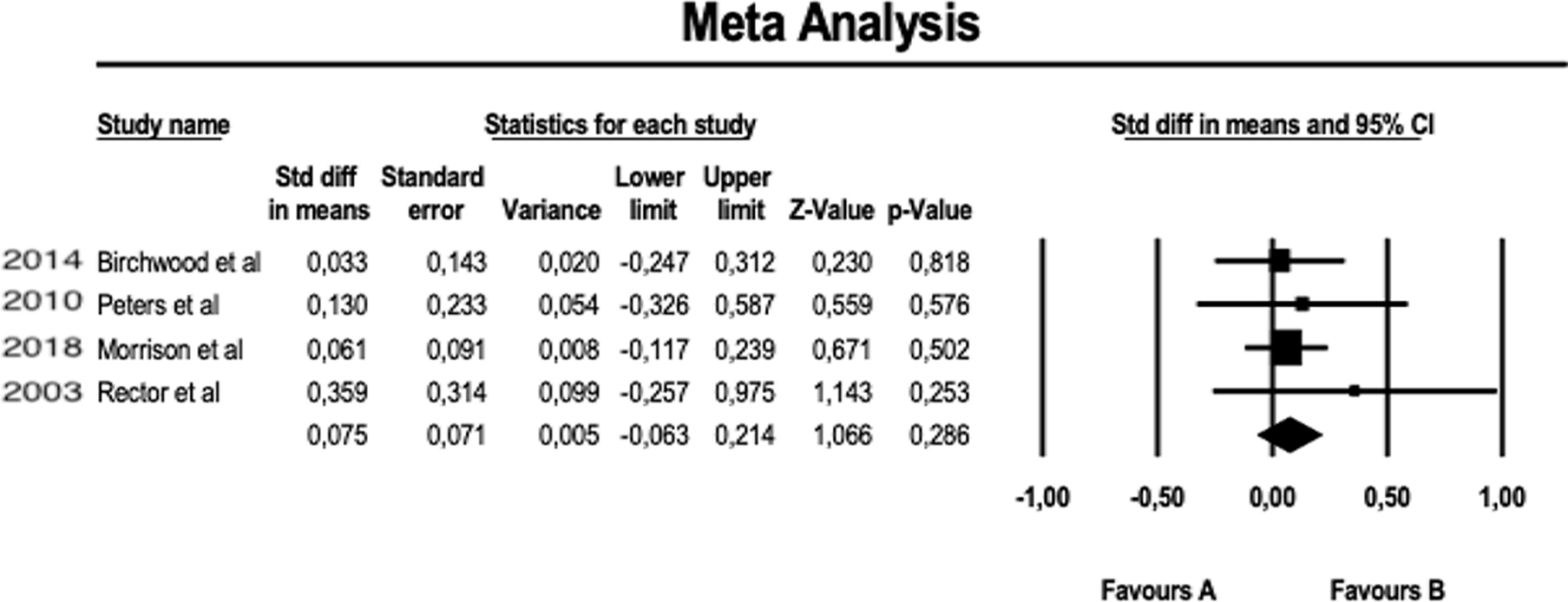
Meta-analysis of PANSS negative symptoms.

**Figure 4:**
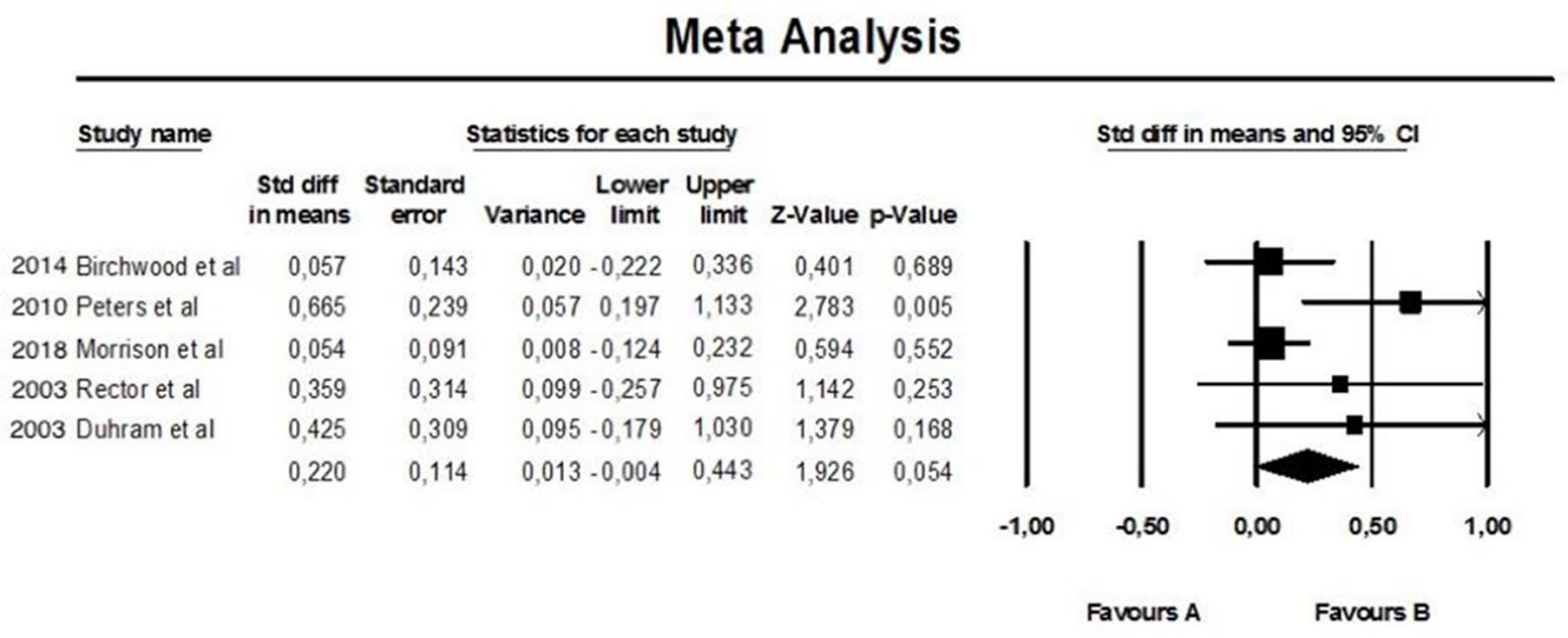
Meta-analysis of PANSS total.

About the meta-analysis portion, we performed a fixed-effect meta-analysis aimed at evaluating the efficacy of augmentation therapy with CBT on the positive symptoms of Positive and Negative Syndrome Scale (PANSS) (see **Figure 2**). The same analysis was replicated on the negative symptoms of PANSS (see **Figure 3**). A further meta-analytical random-effect evaluation was carried out in order to evaluate the effectiveness of augmentation therapy with CBT in terms of variation of the total PANSS scores (see **Figure 4**). The estimate uses SMD (standard mean difference pre- vs. posttreatment) as an effect size.

**Figure 5:**
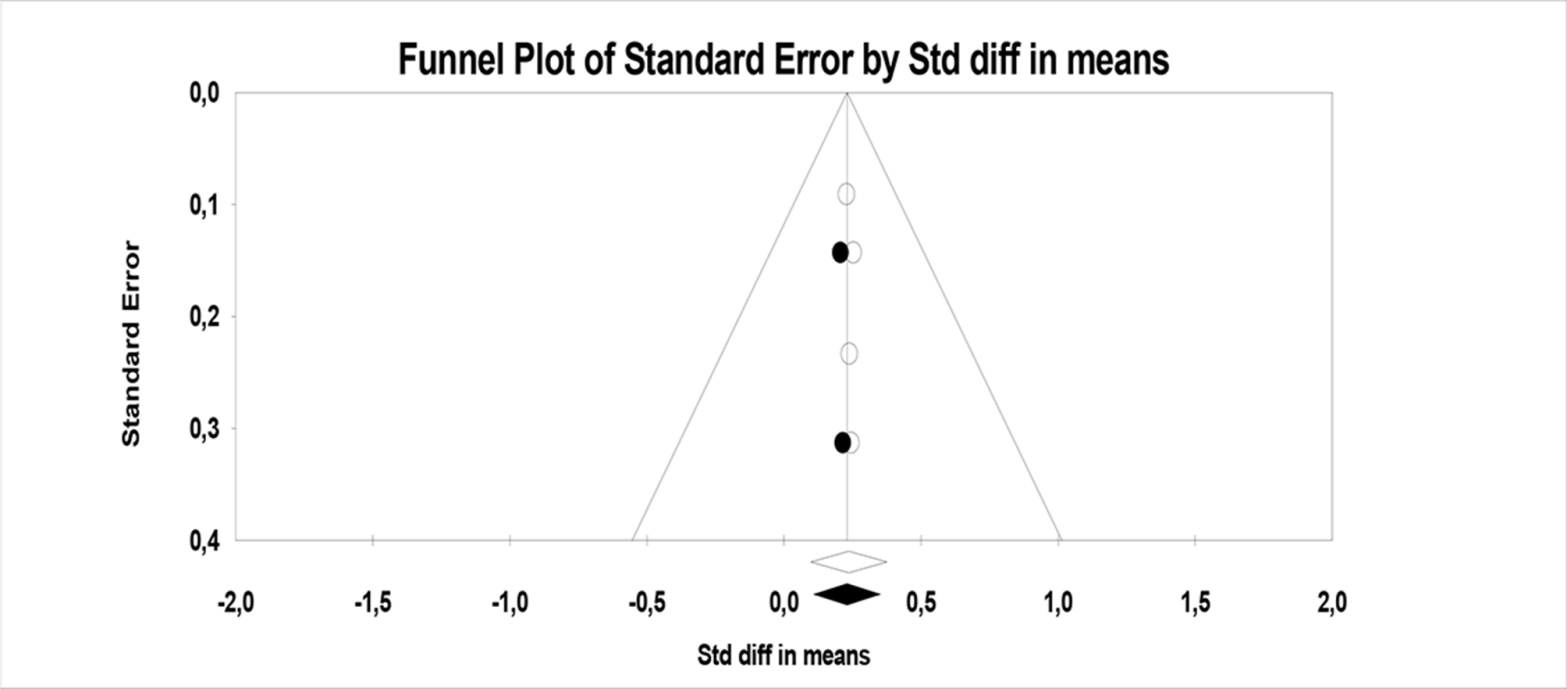
Funnel plot of PANSS positive symptoms.

**Figure 6:**
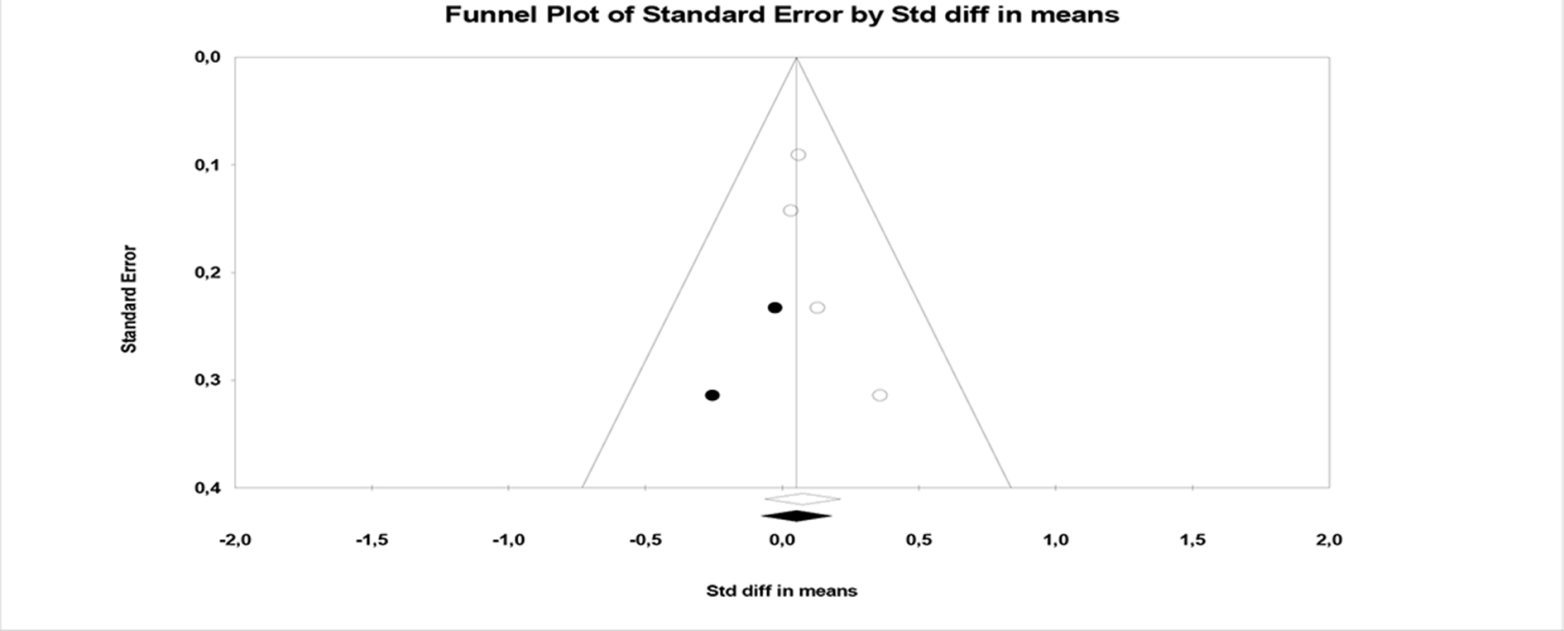
Funnel plot of PANSS negative symptoms.

**Figure 7:**
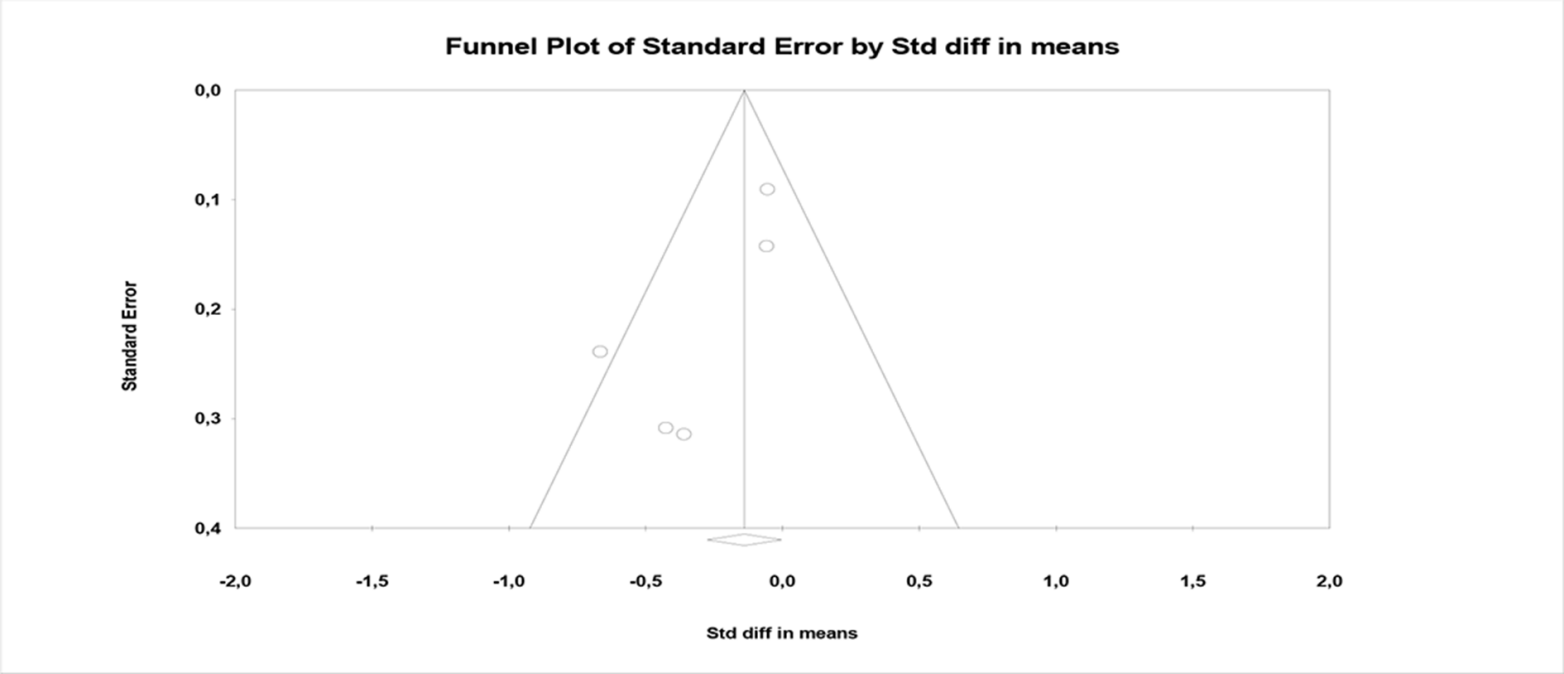
Funnel plot of PANSS total.

The heterogeneity index of the studies and the publication bias were respectively evaluated with *I* ^2^ and Funnel plots (see **Figures 5**, **6**, and **7**).

The inclusion criteria used for the selection of the RCTs suitable for the meta-analysis carried out were as follows:

1. Presence of a uniform control group (patients treated with the usual therapy) (TAU)
2. Measurement of outcome with validated scales (PANSS)
3. Studies only of the RCT type
4. Same type of psychotherapeutic intervention (individual CBT)
5. Evaluation, pre- and posttreatment, with the same type of scale
6. Follow-up to 6 or 9 months

### Study Selection

Included papers were those reporting efficacy outcomes about the positive and/or negative symptoms of TRS and TRP exposed to antipsychotic replacement or augmentative psychotherapy, any modality. Outcome measures could be reordered by means of varying standard rating tools or by means of the clinicians’ judgment.

### Data Collection Process

Two authors (DP and MP) conducted a two-step literature search, examining all titles and abstracts, accessing the full texts of potentially relevant papers. Upon data collection and extraction, the appointed authors compared their results with each other to reach a final consensus based on consensual inclusion and exclusion criteria. Any eventual discrepancy between the principal investigators, blind to each other, was solved by consultation with the senior author (AdB). Finally, the leading senior author with considerable experience on the topic (AdB) assisted in manuscript revision. Data were sought for the following characteristics: participants, interventions, comparisons, outcomes, and study design (PICOS), as well as funding sources. Specifically, the recorded variables for each article included in the review were the following: author(s), year of publication, study design, sample size, eventual follow-up or control group, outcome measures, conclusions, limitations, quality score, and quality differentiation.

### Risk of Bias in Individual Studies

Potential major confounding biases in the studies were ascertained at study level focusing on the following: measurement/diagnostic bias (e.g., lack of reliable diagnostic tools to make the diagnosis of TRS or TRP), confounding bias (e.g., lack of stratification and multivariate control for specific sociodemographic, vital, or clinical features), information (especially recall) bias, unrepresentativeness or inhomogeneity of the sample size or lack of control group (where applicable), and selection by indication bias (nonrandom assignment of the exposure where applicable) (111).

### Scoring and Ranking of the Studies

The present systematic review purposely encompassed a broad range of records and different types of study designs. To avoid an “apples and oranges” bias, we strived at stratifying the appraised results by discriminating between different quality levels. Specifically, observational case–control reports were appraised by means of the Newcastle–Ottawa Rating Scale (see **Table 1**) (118) and randomized controlled studies were appraised using the Jadad scale (see **Appendix 1**) (119).

**Table 1:** Newcastle-Ottawa Scale for assessing the quality of the included studies.

		Newcastle–Ottawa Scale Case–Control Studies (http://www.ohri.ca/programs/clinical_epidemiology/oxford.asp)					
Author	Year	Selection—case definition	Selection—representativeness of the cases	Selection—selection of controls	Selection—definition of controls	Comparability of cases and controls	Exposure/ascertainment of exposure
Ross et al. (112)	2009	*	*	*	*	*	*
Cather et al. (113)	2005	*	*	*	*	*	*
Temple, Ho. (124)	2004	*	*	*	*	**	*
Randal et al. (114)	2003	*	*	*	*	**	*
Durham et al. (115)	2003	*	*	*	*	*	*
Levine et al. (116)	1998	*	*	*	*	*	*
Garety et al. (117)	1994	*	*	*	*	*	*

### Risk of Bias Across the Studies

Any eventual bias affecting cumulative evidence (e.g., publication bias, selective reporting within studies) was assessed through the study evaluation process and accounted in the discussion of the present manuscript.

## Results

The process of the literature search is shown in **Figure 1**. The search identified 42 references, of which 18 were RCT articles (see **Table 2** for all the types of studies). **Appendix 1** provides an overview of descriptive information about the 42 studies.

**Table 2:** Study design of the included trials.

Type of study	Number of studies	Number of studies with blind assessors
RCTs	18	14 blind studies
Randomized experimental trials	1	0 blind studies
Controlled clinical trials	5	2 blind studies
Uncontrolled clinical trials	6	0 blind studies
Case reports	3	0 blind studies
Pilot studies	2	0 blind studies
Follow-up studies	3	2 blind studies
Meta-analysis	3	2 (1 blind study + 1 blind vs. nonblind study)
Cochrane Intervention Review	1	1 blind vs. nonblind study
Total	42	21

### Overall Number, Selected Number, and Typology of Psychotherapy Intervention

Only patients who had been stable on medication for a defined period (from 8 weeks to 6 months) were included in the studies. As reported in **Table 3**, CBT works were found in 32 trials: 25 on individual and 7 on group CBT. Social skill training (SST) was studied in adjunction to CBT, and they were compared to supportive counseling (SC) in one trial (120). Works on family interventions (FI), psychosocial intervention (PI), psychoeducation (PE), key-person counseling (KC), cognitive remediation (CR), supportive counseling (SC), and supportive therapy (ST) were studied in comparison with CBT in 12 CBT works. No studies with these interventions alone on TRP patients have been found. In one trial, CBT was compared to SC plus PE (121). Mindfulness was used in adjunction to CBT, acceptance-based intervention (ACT), and treatment of resistant command hallucinations (TORCH) in one study (122), while it was examined alone in another work (123). One study on multimodal individual psychotherapy, including individual CBT, was found (114). Two controlled trials that compared individual CBT to treatment as usual (TAU) have been collected (117, 124). One RCT that compared CBT to enriched TAU (125) has been found. The studies regarding other interventions alone were as follows: reasoning training (RT, *n* = 2) (112, 126), metacognitive therapy (MCT, *n* = 2) (127), cognitive therapy for command hallucinations (CTCH, *n* = 1) (128), art group therapy (*n* = 1) (129), occupational therapy (OT, *n* = 1) (130), and psychodynamic-interpersonal therapy (PIT, *n* = 1) (131).

**Table 3:** Type of psychological intervention in the retrieved studies.

Psychological intervention	Number of studies
Individual or group CBT vs. treatment as usual	17
and/or other nonspecific therapies	
CBT, Psychosocial Intervention	2
CBT, Supportive Therapy	3
CBT, Psychoeducation (PE)	2
CBT, Supportive Counseling (SC)	1
CBT, SC + PE	1
CBT, Psychoeducation, SC	2
CBT, Family Intervention	1
CBT, Social Skill Training (SST), ST	1
CBT, ACT, TORCH, Mindfulness	1
CBT, Cognitive Remediation (CR)	1
Individual Multimodal Psychotherapy	1
Cognitive Therapy for Command Hallucinations	2
Reasoning Training	1
Mindfulness	1
Metacognitive Therapy	2
Art Group therapy	1
Occupational Therapy	1
Psychodynamic Interpersonal Therapy	1
Total	42

Ten of the 42 studies regarded group therapy (116, 123, 129, 132–138). They are shown in **Table 5**.

The CBT studies represented the majority of articles (32 out of 42). They were generally rigorous, as 22 out of 32 were of the RCT type, including 3 follow-up studies and 2 meta-analyses, while 10 studies included six trials with a control group. Only four CBT studies had no comparison group or control group. Some CBT researchers have used befriending (BF) (122, 139, 140). An RCT study on BF in first episode psychosis has been found and reported in **Appendix 2** (64). The mindfulness study used BF as a comparison group as well. The work on multimodal psychotherapy used a TAU control group. Of the remaining studies, 3 out of 9 included a control group: the brief RT was compared to the Attention Control Activity (141), OT was compared to clozapine alone (130), and the CTCH was compared to TAU (128).

Moreover, a meta-analysis (138) was focused on individual and group FI studies on schizophrenia patients who were both TR and not TR patients and included CBT works in TRS patients, who were accurately described.

For this reason, it has been incorporated in our work. A second phase of the same meta-analysis has been excluded, as it did not pertain to medication resistance (142). The dose of treatment was measured by the total number of sessions and was from 4 to 27, given throughout a period between 12 weeks and 24 months. In five studies, the number of sessions and the time of treatment were not specified.

### Therapists and Blindness

Therapists were generally expert, except for one case (143). In two cases, the raters were trained and experienced nurses (140, 141). One study specifically on treatment resistance in early psychosis was found (144). Another study included early psychosis in a heterogeneous group (93). Eighteen articles were trials with blind raters, while blindness could not be used in 21 works. Only one meta-analysis out of three was specifically focused on blind studies (145). The Cochrane review compared blind studies with nonblind studies (146).

### Stage of Illness

The stage of illness (initial or chronic) was heterogeneous in 10 studies, where the patients who were enrolled had different ages or very diverse duration of illness. In 11 articles, the duration of illness was not specified. Twenty trials had a sample of chronic TRP patients. None of the found articles resulted in reporting the DUP.

### Pharmacological Co-Treatment

In only two studies were there patients who were not on medication. In the first, there were 5 of 40 patients in an uncontrolled naturalistic study of CBT plus FI (147), while in the second there were 3 of 12 patients on individual CBT in a RCT (148). Remarkably, no study has proposed a psychological treatment as an alternative to medication in the whole sample. No study with regard to music therapy, specifically on medication-resistant psychosis patients, has been found. However, other 18 studies, which did not focus on TR patients and were not included in this research, have been collected in **Appendix 2**.

### Clinical Outcome

Assessments used to measure improvement often differed between the various trials. Hence, we pooled study results either based or not based on statistics along with the authors' conclusion to compare them. See **Table 4** for details on the number of works that had statistically significant outcomes. Articles reporting no improvement are also included in **Table 4**. No changes after treatment have been observed in only 2 studies out of 42. Those two trials were on CBT: one on CBT integrated with FI (147) and one on group CBT (132).

**Table 4:** Improvements observed in the different psychological interventions, which were examined in the reviewed studies.

Psychological intervention on TRP patients	Studies with statistically significant improvement	Studies with no statistically significant improvement	Studies with no different improvement between groups	Studies with no improvement	Studies with improvement specifically on negative symptoms
Individual CBT Individual CBT vs. Befriending Group CBT Group CBT vs. Group ST CBT vs. Psychosocial Intervention CBT, Supportive Therapy CBT vs. Psychoeducation CBT vs. Supportive Counseling CBT, Family Intervention CBT, Social Skill Training vs. ST CBT, (ACT, TORCH), Mindfulness vs. Befriending CBT vs. Cognitive Remediation Multimodal Psychotherapy Reasoning Training Cognitive Therapy for Command Hallucinations (CTCH) Mindfulness Metacognitive Therapy Art Group Therapy Occupational Therapy Psychodynamic Interpersonal Therapy	8 1 3 1 0 0 0 2 1 0 0 0 1 1 1 0 2 0 1 0	2 0 0 0 0 0 0 0 0 0 0 0 0 1 0 1 0 1 0 1	0 1 0 1 1 1 2 3 0 1 1 1 0 0 0 0 0 0 0 0	0 0 1 0 0 0 0 0 1 0 0 0 0 0 0 0 0 0 0 0	2 1 0 0 0 0 1 0 0 0 1 0 0 1 1 0 1 0 1 1
Total	22	6	12	2	10

### Symptoms and Clinical Domains

The symptomatology studied in the retrieved trials is mainly represented by the positive symptoms and, above all, the auditory hallucinations, especially in the CBT studies, while negative symptoms have been rarely evaluated. Ten studies out of 42 reported a decrease of negative symptomatology (see **Table 4**). Efficacy on negative symptoms has been shown in three CBT trials and in ST, CR, CTCH, and OT. Art group therapy, MCT, and PIT trials have also reported a positive outcome on negative symptoms but without control group and not statistically evaluated (see **Appendix 1** and **Table 4**). Affectivity has not been specifically evaluated, except for art group therapy and PIT. Clinical progress has also been observed in other areas, such as social functioning and personal care. Self-esteem and hopelessness have been evaluated, but their improvement has not been shown.

Studies with chronic patients affected by treatment resistance have shown that CBT could be effective, providing positive symptom reduction, which was considered equivalent to a “medium effect size.” A trend to effective treatment has been observed as well, in case series with psychosis onset, which was resistant to medication alone: almost three-quarters of patients achieved clinically significant improvement (144). However, results of CBT efficacy compared to other treatments in TRP are not homogeneous in all studies. For instance, when compared to other treatments, similar improvements to the CBT experimental group have been observed in other comparison groups, while a significant difference has been constantly observed only from TAU (132). In particular, in the Cochrane meta-analytical review on schizophrenia including TRS, psychosocial therapies have shown no clear difference from CBT for outcomes relevant to adverse effect/events, global mental state measures, and effects on positive or negative symptoms (146). Moreover, the studies comparing CBT to another treatment, such as cognitive remediation (149), befriending (122, 140), supportive therapy (115, 120, 134), psychoeducation (113, 150), supportive counseling (121), or family intervention (138), have shown significant clinical improvement in all groups that were studied. Finally, a statistically significant major improvement in supportive therapy has also been observed (135). Two trials have shown a significant improvement in the CBT group when compared to other psychological interventions, such as befriending (139) or supportive counseling (151). Moreover, in two follow-up studies, CBT did not maintain the superiority to SC (152, 153). In particular, after 1 year from the end of treatment, CBT started to decline while SC improved, and this trend continued at 2-year-follow-up. Finally, our results show that group therapy is related to significant improvement for all psychological interventions retrieved, except for family intervention (138), where single family treatment resulted better than the group family one. In six out of seven trials, group CBT presented the same improvement as the comparison group, showing the same results that were observed in the studies on individual CBT.

### Meta-Analysis Result

The results obtained in our meta-analysis concerning the domain “POSITIVE SYMPTOMS” of the PANSS scale are as follows:

Fixed-effect meta-analysis: number of studies = 4; number of comparisons (*k*) = 4; total sample = 800 patients; SMD (standard mean difference) = 0.237 (C.I. = 0.097–0.376).

These preliminary results suggest that, on average, the PANSS score for positive symptoms was reduced by 23.7% more (with a margin between 9.7% and 37.6%) in patients who performed augmentation therapy with CBT compared to patients who received the usual therapy (TAU) (see **Figure 2**). Moreover, this reduction is statistically significant (*p* = 0.001).

Although the number of meta-analyzable studies is small, the heterogeneity index *I* ^2^ is 0% (**Figure 5**).

The results obtained in our meta-analysis concerning the domain “NEGATIVE SYMPTOMS” of the PANSS scale are as follows:

Fixed-effect meta-analysis: no. of studies = 4; number of comparisons (*k*) = 4; total sample = 800 patients; SMD (standard mean difference) = 0.075 (C.I. = −0.063–0.214).

These preliminary results suggest that, on average, the PANSS score for negative symptoms was reduced by 7.5% more (with a margin between −6.3% and 21.4%) in patients performing augmentation therapy with CBT compared to patients receiving the usual therapy (TAU) (see **Figure 3**). However, this reduction is not statistically significant (*p* = 0.286).

Furthermore, it is noteworthy that the “lower limit” of the negative confidence interval (−6.3%) indicates how, at least in a small number of events, the CBT in augmentation to the usual treatment (TAU) could potentially induce even an effect opposite to the therapeutic one.

Although the number of meta-analyzable studies is small, the heterogeneity index *I* ^2^ is also 0% in this case (**Figure 6**).

The results obtained in our meta-analysis concerning the “TOTAL Score” domain of the PANSS scale are as follows:

Random-effect meta-analysis: no. of studies = 5; number of comparisons (*k*) = 5; total sample = 843 patients; SMD (standard mean difference) = 0.220 (C.I. = 0.443–0.004).

These preliminary results suggest that, on average, the total score at the PANSS was reduced by 22% more (with a margin between 44.3% and −0.4%) in patients who performed augmentation therapy with CBT compared to patients who received the usual therapy (TAU) (see **Figure 4**). However, this result is not statistically significant (*p* = 0.054).

Moreover, in this case, the heterogeneity index *I* ^2^ is equal to 46% and, being quite high, therefore indicates a poor homogeneity of the analyzed data (**Figure 7**).

## Discussion

### Psychological Interventions

Psychological interventions in TRP patients have shown a therapeutic effect in 40 out of 42 selected studies. In particular, results demonstrate improvement in positive symptoms for CBT, as well as for other psychological interventions, albeit with different degrees. More specifically, CBT effects in selected studies were not statistically different respectively from psychosocial intervention (146), cognitive remediation (149), befriending (122, 140), supportive therapy (115, 120, 134, 135), psychoeducation (113, 150), supportive counseling (121), and family intervention (138).

CBT has been recognized as more efficient in persistent positive symptoms at follow-up. Supportive counseling (SC) was less effective than CBT at the 9-month follow-up, while it demonstrated the same efficacy as CBT at the following follow-up. Finally, the SC showed its superiority in some measures at 2 years follow-up (140, 153). It has been speculated that supportive counseling may enhance frequent and regular nonthreatening social interaction, which might have worked on self-esteem and helped patients to recuperate their social activity (16). Furthermore, metacognitive therapy has also shown significant improvements in both positive and negative symptoms compared to the baseline (but a control group was not provided) (127). Although art therapy is not strictly considered as a form psychotherapy, it has shown to lead to improvements in a short time in fields that are not easily measured by regular assessments, for example when considering interhuman relationship (129). Moreover, affectivity has not been specifically evaluated, except for art group therapy and psychodynamic interpersonal therapy (131), which were case series. In this work, clinical progress has also been observed in other areas such as social functioning, showing a marked reduction in the severe disturbances presented prior to treatment (131). Occupational therapy has been shown to give a statistically significant improvement compared to clozapine alone in the performance of the activity, in psychotic symptoms, social interaction, and personal care (130). Multimodal psychotherapy, reasoning training, and cognitive therapy for command hallucinations (CTCH) have also shown significant improvements compared to TAU (112, 114, 126, 128). The sample population targeted in the trials included different phases of the illness, showing that an *integrated* treatment with psychological intervention and pharmacological treatment could be helpful at any point of the disease trajectory. On the contrary, no data on the use of psychological intervention alone on TRP patients are currently available.

Few methodological issues need to be considered, such as the *type of intervention*, *characteristics of the sample*, including *age of patients* as well as *stage of the illness*, and *duration of the treatment*. With regard to the type of intervention, it has already been observed that all psychological therapies, including befriending and supportive therapy, may have a clinically relevant impact, and statistically significant results are reported in more than half of the trials included in this review (22 out of 42, see **Table 4**).

A controversial aspect of psychotherapeutic interventions in TRP is represented by the fact that psychological interventions, including CBT, have an effect mainly on positive symptoms while they seem to be less clearly effective on other main aspects, such as negative and cognitive symptoms. Eighteen CBT trials have shown that CBT, in adjunction to antipsychotics, could produce better outcomes on a variety of measures than medication alone, but target treatment was mainly represented by positive symptoms. In fact, negative symptoms are generally left aside and remained prevalently persistent in the majority of studies. In some trials, negative symptoms have not even been evaluated. In summary, 10 studies out of 42 reported a significant reduction of negative symptomatology: 3 on CBT, 1 on CR, 1 on MCT, 1 on CTCH, 2 on occupational therapy, 1 on art group therapy, and 1 on psychodynamic interpersonal therapy (see **Table 4**). These evidence are also compatible with the result of a recent meta-analysis on psychological treatments of negative symptoms in a population of psychotic patients that were not specifically resistant to treatment (154). In particular, improvement in negative symptoms has been observed after CBT intervention in patients who were at any stage of the disease. This amelioration has resulted in 59% of the studies when CBT was compared to TAU, while none of the analyzed studies suggested a benefit of CBT if compared to active controls. Moreover, another recent meta-analysis for a total of 4,068 patients who were on average moderately ill at baseline has confirmed the efficacy of CBT on positive symptomatology (155). A recent systematic review has newly reported that CR can also have beneficial effects on negative symptoms, compared to TAU and TAU plus active control in schizophrenia patients who were not treatment resistant (68).

Additional researches are needed in order to test “self-disturbance” (156, 157). Consequently, it would be necessary “to tailor” psychological treatment aimed at this symptom. Since the “hyperreflexive attitude” is typical in self-disturbance and in nonaffective psychosis, CBT might not be the most suited psychological intervention on these patients. This is due to the fact that an important feature of this therapeutic approach is the encouragement of “thinking about thinking” (14, 158), which is what the patients already do repeatedly in a pathological fashion (159).

It has been observed that brain dysfunctions, for example, dopaminergic supersensitivity, could be secondary to psychological events (74, 160). Furthermore, studies on brain receptor availability after psychotherapy treatments (both CBT and psychodynamic psychotherapy) have shown that a neurobiological alteration can be modifiable or reversible thanks to psychological interventions (161–164). Further steps in augmentation with psychological therapy of TRP seem to be focusing on the total symptomatology, including positive, negative, and self-disturbance. Considering that symptoms are part of unitary and complex psychopathology, acting on one aspect could be partial. On the other hand, publications on psychodynamic psychotherapy, which is focused on unconscious dimension, are poorly available; only one paper referring specifically to TRP patients has been found in this review (131).

Other critical points are as follows: the characteristics of the sample, age of patients, stage of illness, and duration of the treatment. Some gaps have to be highlighted. Firstly, a marked heterogeneity of the selected sample has been observed in 10 trials, while 11 studies did not take it into account. For instance, patients at different ages or at difference stages of illness (early stage, acute or chronic phase) were located in the same group. For example, 18-year-old patients were in the same group as 40-, 50-, and 60-year-old patients: considering the different psychopathological conditions and the long-term effects of the illness (165), patients respond differently.

Furthermore, it has been observed that factors associated with better outcome include a shorter duration of illness and less severe symptom at pretreatment (151, 166). In addition, in the acute phase of psychosis, CBT can produce durable and substantial clinical benefits (165). Concerning the detailed diagnosis of TR, if two different types of TRP or TRS (at the early and at the chronic stage of illness) have been identified, they should be studied separately and not in the same sample. Secondly, in the majority of studies, the duration of the treatment ranged from 4 weeks to 9 months. Only 2 studies out of 42 (114, 138) used a duration of treatment up to 21–24 months, and in one study (147), the length of intervention was 12 months. In two studies, therapy was administered in one single session (112, 126), and in four trials, duration of treatment was not even specified. A significant recovery could not be expected during a 2-month treatment period, when patients are markedly ill and/or chronic with persistent and expressed negative symptoms of schizophrenia (129). This is supported by the observations of an increased effect over time of CBT on mental state (140). For instance, in the selected articles, a longer duration of treatment can generally show better results on negative symptoms. On the other hand, recent publications on the comparison between short- and long-term psychotherapy have shown contrasting results (167, 168). However, these works were referred to nonpsychotic patients. A short-term duration is insufficient for psychotic onset patients, who need to be treated longer, considering guidelines (169). Finally, according to our results, as reported in **Table 5**, group therapy should also be encouraged, as it is generally well supported by evidence in improving persistent positive symptom in both CBT and other psychological interventions.

**Table 5:** Comparison between different group psychotherapies.

Author/type of study	Efficacy	Comparison between different group therapy	Type of therapy
Mandić-Gajić G (129) Case reports	Yes	No	Group art therapy
Jacobsen et al. (123) Uncontrolled study	Yes	No	Group Mindfulness
Penn et al. (135) RCT	Yes	Yes, improvement in ST at posttreatment and in both groups at follow-up	Group CBT, Group ST
Johnson et al. (134) RCT	Yes	Yes, improvement in both groups with no significant difference	Group CBT, Group ST
Barrowclough et al. (132) RCT	No	No	Group CBT
Wykes et al. (137) RCT	Yes	No	Group CBT
Pinkham et al. (136) Pilot study	Yes	No	Group CBT
Pilling et al. (138) Meta-analysis (part of the study including heterogeneous population: both TRP and not TRP)	No	No. No comparison has been made with single FI. Single FI became more efficient than group FI (not statistically significant)	Group FI (vs. Individual CBT)
Chadwick et al. (133) Uncontrolled study	Yes	No	Group CBT
Levine et al. (116) Controlled trial	Yes	No	Group CBT

### Exploratory Meta-Analysis of Cognitive Behavioral Therapy Interventions

The results obtained from our meta-analytical extraction have confirmed that cognitive–behavioral psychotherapy is very effective particularly in the treatment of positive symptoms in TRS and/or TRP patients. This result is in line with what has already been found in other studies in the literature. The same efficacy was not found in the treatment of negative symptoms while it was only partial in achieving an improvement in the total scores of patients evaluated in the PANSS. We have also found that CBT in augmentation with the usual treatment (TAU) works well in the initial stages and then gradually loses effectiveness (170). In this regard, we can hypothesize that schizophrenia worsens over time, making treatment with CBT more difficult and therefore less effective; moreover, it could happen that, in the initial stages of treatment, there is a sort of “feeling of well-being” that does not necessarily coincide with a real clinical improvement. However, there are very few studies with a sufficiently long follow-up to clarify these hypotheses. As regards the low incisiveness of CBT on negative symptoms, we can hypothesize that patients with more pronounced negative symptoms and therefore with affective dullness and social withdrawal are less suitable for this type of psychotherapeutic approach or that these symptoms require a longer duration of treatment to be effectively affected. Moreover, given that the few studies in the literature with a longer follow-up have shown an efficacy also on the negative symptoms, we can hypothesize that the patients followed for a longer period may have benefited from therapeutic adjustments over time as well as from the CBT. The limits of these results are in some way superimposable to those already listed above about the systematic review on the same topic. In addition to what has already been said, the incompleteness and the partiality of the data at our disposal are worth noting, as, for example, not all the articles indicated the dropout rates accurately, or at what time of the treatment they occurred, or which group they belonged to (cases or controls).

## Conclusions and Future Research Directions

Psychotherapy should be considered a potential relevant therapeutic strategy in adjunction to medication in TRP patients. An intervention on psychosis that does not consider an integrative approach could miss a potential effective component of the treatment. However, few questions need to be addressed in the future in order to better understand the role of psychotherapy in TRP. Firstly, it would be appropriate to start with large-scale multicenter, controlled studies based on psychotherapeutic approaches (i.e., CBT) that were shown to be effective in smaller studies and to include patients with homogeneous domains of symptoms, duration and doses of antipsychotic treatment, as well as duration of illness. Secondly, a longer time of treatment should be conceived in such studies in order to get an adequate signal of the response. Finally, even if challenging, an important issue is to consider the inclusion of biological markers (i.e., functional imaging) before and after the introduction of the psychotherapeutic augmentation or of the substitution psychotherapy. Moreover, future studies need to adopt reliable operational outcome measures for non-CBT studies to allow quantitative extraction of information and reliable comparison of efficacy measures for psychological interventions other than cognitive therapy that are currently almost invariably not assessed in a controlled, RCT fashion.

## Author Contributions

DP designed the study, searched the database, wrote the article, and created the appendices, tables, and figures. MP searched the database and participated in the editing of the manuscript. MF and VD supervised the literature procedure extraction, commented on the last draft, and contributed to the writing of the manuscript. AdB wrote and commented the manuscript, as well as supervised all work including the design of the study and the final draft. All authors have read and approved the final version of the manuscript.

## Funding

The Open Access has been supported by a grant of the Department of Neuroscience, Reproductive Science and Odontostomatology of the University of Naples “Federico II” to the Section of Psychiatry (AdB and FM).

## Conflict of Interest Statement

AdB has received research support from Janssen, Lundbeck, and Otsuka and lecture honoraria from Chiesi, Lundbeck, Roche, Sunovion, and Takeda; he has served on advisory boards for Eli Lilly, Janssen, Lundbeck, Otsuka, Roche, and Takeda. The remaining authors declare that the research was conducted in the absence of any commercial or financial relationships that could be construed as a potential conflict of interest.

## Appendix 1

**Table:**

Author/type of study	Psychological intervention	Adjunction/antipsychotic	Number of patients/comparison groups	Stage of illness/age of patients/diagnosis	Frequency (and time) of sessions/duration of treatment	Results	Jadad score
Morrison et al. (171) Uncontrolled trial Nonblind study	Metacognitive therapy (MCT)	Yes/atypical	10 patients MCT/none	Not specified/34.3 years+/schizophrenia (SKP), schizoaffective disorder (SKA), delusional disorder (DD), other*	6–12 sessions/9 months	PANSS total and positive significantly reduced at the end of treatment (E) and at 3 months follow-up (FU) PSYRATS reduction with borderline significance	–
Hutton et al. (127) Case report randomized nonblind study	MCT	Yes/various	3 patients MCT/none	Chronic SKP/15, 20, and 40 years/KA, DD, with positive symptoms	11–13 1-h weekly sessions/3 months	Clinical worthwhile benefits in all. In 2 patients, significant PANSS reduction and increased recovery. At 3 months, FU reduction in positive and negative symptoms	–
Birchwood et al. (93) RCT Single blind	Individual CBT for command hallucinations	Yes/atypical	98 patients CBT+ Treatment as usual (TAU)/99 TAU	Heterogeneous/≥16 years/SKP, SKA, psychosis (P), bipolar disorder (BD), with self-harm	Up to 25 sessions/9 months	Reduction of compliance to voices but not significant	3
Burns et al. (145) Meta-analysis Blind study	Individual CBT	Yes/various	552 patients/waiting list, TAU or other	Not specified/not specified/SKP, SKA, DD	10–24 sessions/6 weeks to 9 months	Statistically significant beneficial effects of CBT at E and FU for positive and general symptoms	–
Jones et al. (132) Cochrane Intervention review Blind vs. nonblind studies	CBT vs. other psychosocial therapies	Yes/various	Not specified; 20 trials/CBT/psychosocial therapies/TAU	Heterogeneous/18–65 years/SKP	Various	No advantage for CBT over other treatments, including less sophisticated therapies	–
Mandi´c-Gaji´c G (129) Case report Blind study	Group art therapy	Yes/various	2 patients/no control group	Chronic/31 years, 27 years/paranoid and simplex SKP with severe negative symptoms	Not specified/2 months	Improvement in all symptoms, but not statistically significant. Intervention has helped to understand the inner world of patients	–
Klingberg et al. (149) RCT Blind study	Individual CBT, cognitive remediation (CR)	Yes/not specified	99 CBT+TAU/99 CR+TAU	Chronic/18–55 years/SKP outpatients at least with moderate negative symptom	16.6+ CBT sessions vs. 13.7+ CR sessions/9 months	No suicide, at the E and at 3 months FU adverse events (AEs) in 10 CBT patients, including suicidal attempts, and in 5 CR patients. Depression more frequent in CBT patients. Not statistically significant results	3
Shawyer et al. (122) RCT Blind study	CBT with acceptance-based intervention (ACT), treatment of resistant command hallucinations (TORCH), mindfulness, befriending (BF)	Yes/not specified	21 CBT/21 CBT/22 BF/17 control group (waiting list)	Chronic/18–65 years (39 years+)/SKF (72%), SKA (21%) and affective P (7%)	1,5 50-min weekly sessions/4–6 months. 10 minutes mindfulness exercises, with home practice	Subjective greater improvement in CBT vs. BF, but not significant results. CBT here has more modest effect that in early studies	4
Waller et al. (126) Pilot study Nonblind study	Reasoning training Maudley review training program	Yes/not specified	13 patients/no control group	Chronic/44.6 years (mean)/P with low levels of belief flexibility, with jump to conclusion	Single session. One-off computerized training package, lasting approximately 1.5 h	Significant improvement at post-intervention in belief flexibility and improved reasoning	–
Erickson (144) Uncontrolled study Nonblind study	Individual CBT	Yes/according to the early psychosis program	14 patients/no control group	Early psychosis patient/≥18 years/SKP spectrum outpatient	15–25 sessions/not specified period	Significant reduction of positive symptoms and not significant reduction of PANSS negative scale	–
Peters et al. (143) RCT Nonblind study	CBT by nonexpert therapists (supervised)	Yes/various; unmedicated: 6% in the therapy group, 3% in the control group	36 CBT (in 2 CBT groups)/38 TAU	Not specified/18–65 years/P with persistent positive symptoms	16 (mean) weekly or forthrightly sessions lasting up to 1 h/6 months	Significant main result in depression, in CBT. At PANSS, positive improvement only in one CBT group	3
Jacobsen et al. (109) Uncontrolled study Nonblind study	Mindfulness Group	Yes/Not specified	8 patients/No control group	Chronic/ 21–43 years/Complex Psychosis inpatients	1-hour weekly session with 3–5 people/6 weeks	Improvements in PSYRATS, SMQ and a stress scale, but a statistical analysis of results was not provided	
de Paiva Barretto EM et al. (139) RCT Blind study	Individual CBT, befriending (BF)	Yes/clozapine	12 CBT/9 BF	Chronic (CBT 15++ years, BF 10++ years)/CBT 39.8+ BF 33.2+/TRS	20 sessions/21 weeks	Statistically significant improvement in positive symptoms in CBT. Reduced negative symptoms in both groups but not significant	1
Penn et al. (135) RCT Blind study	Group CBT/group supportive therapy (ST)	Yes/at least two trials, one atypical for 8 weeks prior to randomization	32 group CBT/33 group ST	Non specified/18– 65 years/SKP or SKA outpatients	Twelve 1-h weekly CBT sessions/3 months; 12 weeks of enhanced ST (172)	Statistically significant improvement only in ST group at E. At 12 months FU significant reduction also in CBT group at PANSS. ST had more specific impact on hallucinations	4
Brabban et al. (141) RCT Blind study Nurses	Brief CBT	Yes/not specified	226 CBT/128 TAU	Not specified/CBT 40+, TAU 41.2+/SKP	From three to six 1-h sessions/2–3 months	Improvement, but not significant	3
Ross et al. (112) Randomized experimental trial Nonblind study	Reasoning training (RT), attention control activity (ACA)	Yes/not specified	1st stage: 34 RT/34 healthy volunteer 2nd stage: 17 RT/17 ACA	Chronic/16.2 years+ RT, 10.8 years+ controls/SKP spectrum disorder	45-min reasoning intervention in 3 tasks	After training, 24% showed greater belief flexibility and 18% showed a reduction in delusional conviction. Not statistically significant.	–
Johnson et al. (134) RCT Blind study	Group CBT/Group ST	Yes/2 trials, one of which atypical for 8 weeks	58 patients randomly assigned to either group	Chronic/42.1+ years/SKP, SKA outpatients	Twelve 1-h weekly sessions, 1–2 therapists for 4–7 patients over 12 weeks	No difference in ratings between groups.	3
Barrowclough et al. (132) RCT Nonblind study	Group CBT	Yes/not specified	12 patients CBT group/12 TAU	Not specified/age of illness: 13.67++ years; 18–55+ years/SKP, SKA	18 sessions of 2 h including breaks over 6 months	No difference between groups at PANSS, SFS, HADS, BHS, RSE, GAF	1
Cather et al. (113) Controlled trial blind study	Functional CBT (fCBT), psychoeducation (PE)	Yes/olanzapine	15 CBT/15 psychoeducation (PE)	Not specified/age of illness: 24.88++ years; 18–65+ years/SKP, SKA	16 weekly sessions over 4 months	Greater benefit for fCBT on positive symptoms at PSYRATS voices subscale. Not statistically significant	–
Zimmermann et al. (173) Meta-analysis Blind studies vs. nonblind studies	CBT (mainly individual)	Yes/various	1484 patients in 14 studies with at least one CBT group with a control group	Heterogeneous; 10 studies on chronic condition and TRP plus 3 studies on acute; 36.02+ years/SKP spectrum	Weekly sessions/5 weeks to 9 months	Significant reduction of positive symptoms in CBT	–
Wykes et al. (137) RCT Blind study	Group CBT	Yes/typical and atypical antipsychotic	45 CBT + TAU/40 TAU (10 people had specific individual psychotherapy, contaminating the sample)	Chronic/39.7+ years/SKP	Seven sessions/10 weeks	Significant improvement for group CBT patients in social functioning at FU. (Effects could be influenced by extra psychological help and change of medication). No improvement in the severity at PSYRATS	4
Valmaggia et al. (121) RCT Nonblind study	Individual CBT supportive counseling (SC) plus psychoeducation (PE)	Yes/atypical antipsychotic	36 CBT/26 SC, PE	Chronic/18–70+ years/TRS	16 1-h sessions: 12 weekly sessions, 3 fortnightly sessions and last session after 4 weeks/22 weeks	No significant differences between the group at PANNS and PSYRATS, except for the factor 2 of the hallucination subscale	2
Pinkham et al. (136) Pilot study	Group CBT	Yes/atypical antipsychotics	11 patients in two CBT groups/	Chronic/39.6+ years/SKP, SKA inpatients	1-h weekly sessions/7 weeks, 11 weeks	Significant changes in both groups in the participants’ beliefs	
Nonblind study			No control			Reduction at PANSS and PSYRATS, but not significant	–
Trower et al. (128) Single-blind RCT	Cognitive therapy for command hallucinations (CTCH)	Yes/typical or atypical	18 CHTC+ TAU/20 TAU	Heterogeneous; 17–60+ years/SKP spectrum disorder with “severe commands”	On average 16 sessions/6 months	Significant reduction of the compliance with voices with maintained results at 12 months FU. Small reduction in negative symptoms	4
Temple and Ho (124) Controlled trial Nonblind study	Individual CBT	Yes/not specified	8 CBT/9 TAU	Not specified. Age of illness onset 21+ years CBT, 24.2+ TAU, 28.8+ years CBT, 35,9+ years TAU/SKP	19–20 sessions/not specified timing and frequency	CBT showed a statistically significant decline in delusions and hallucinations. Trend of reduction in negative symptoms ( *p* = 0.06)	–
Randal et al. (114) Controlled trial Nonblind study	Individual multimodal psychotherapy (individual, flexible and recovery-focused)	Yes/minimum dose of atypical antipsychotics	9 Multimodal psychotherapy + TAU/12 TAU group retrospectively considered	Chronic/age of onset 18.9–19.3 years; duration of illness 8.6–11.2 years; 29–30+ years/SKP, SKA inpatients (rehabilitation)	15 min to 1 h, twice weekly, reduced to weekly and to fortnightly or monthly/up to 21 months	Clinically significant improvements in the overall PANSS, as well as scores for deviant behavioral RCS	–
Rector et al. (125) RCT Blind study	Individual CBT + ETAU (enriched TAU)	Yes/typical, atypical antipsychotic, anti depressants	24 CBT + ETAU/18 ETAU	Chronic/age of onset 21+ CBT-ETAU, 19.2+ ETAU, 37.5+ years CBT-ETAU, 41.2+ ETAU/SKP	20 sessions/6 months	Significant effects for positive, negative, and overall symptom severity at E, but nonsignificant reduction of negative symptoms at 6 months FU	4
Durham et al. (115) Controlled trial Blind study	Individual CBT, supportive psychotherapy (SPT)+	Yes/typical or atypical antipsychotics	22 CBT/23 SPT + TAU/21 TAU	Chronic/duration of illness 15++ CBT, 14++ SPT;10 TAU; 36+ years/SKP, SKA, DD	Up to 20 sessions of approximately 30 min/over 9 months	Significant improvement in CBT and SPT groups vs. TAU, at 3 months FU, but nonsignificant differences between CBT and SPT at E	–
Buchain et al. (130) RCT Nonblind study	Occupational therapy	Yes/clozapine	14 Occupational therapy/12 clozapine	Chronic/age of onset 20.9+ years, 19.67+ years; 33.71+ years, 36.58+ years/TRS	Nonspecified sessions/6 months	Statistically significant difference at EOITO	2
Pilling et al. (138) Meta-analysis RCT Nonblind study	Family intervention (FI) or CBT	Yes/various	1,467 of 18 FI trials/other treatments or TAU or no control group; 528 of 8 CBT studies/several other treatments	Chronic/Duration of illness: 6 ± 3++ years FI, 11+ + years CBT; 31.2+ years FI, 33.9 years CBT/SKP	8 sessions over a short time period, fortnightly for 2 years, then monthly for 4 years for FI; weekly, monthly sessions 6 weeks to years for CBT	Significant benefit more in FI than standard care, nonsignificant when compared to other treatments More improvement in single FI than in group FI. CBT shows clear positive effects at 9 months FU	–
Wiersma et al. (147) Uncontrolled naturalistic study Nonblind study	CBT and coping training in an integrated single family treatment program	Yes/typical (65%) or atypical (8%) antipsychotics, antidepressants, benzodiazepines, and/or other medication (17%); 5 patients used no medication at all	40 patients/no control group	Heterogeneous/duration of auditory hallucinations: 8+ years; 37+ years/SKP	Average number of contacts 15 (varying from 2 to 51)/1–32 months	Worsening or no improvements at PANSS Subjective improvement at the end of the study and at 2 or 4 years FU in patients and family Statistically significant reduction at <2 years, but not at 4 years Disappearance of hallucination in 18% of patients. At discharge, 20% of the patients left without any antipsychotic medication	–
Tarrier et al. (153) 2 years follow-up [Tarrier et al. (151), RCT]	CBT, SC Individual	Yes/various	CBT/SC/TAU At this FU, 61 out of 72 patients were available	See Tarrier et al. (151)	See Tarrier et al. (151)	No significant differences During the 2nd year, CBT continued to decline, whereas SC improved	–
Sensky et al. (140) RCT Blind study Nurses	CBT, befriending	Yes/various	46 CBT/44 BF	Chronic/duration of illness: 14–15++ years; 39–40+ years/SKP	18 45-min weekly sessions/9 months and less frequent after	Significant clinical improvement in both groups at E	3
Davenport et al. (131) Two case reports Nonblind study	Interpersonal therapy (conversational, model of Hobson)	Yes/various	2 patients/no control group	Chronic/onset at 18 years; F, 38 years; M, 43 years/SKP	Weekly community group, twice daily staff handover meetings	Improvement at the Krawiecka Goldberg Vaughan scale for schizophrenia social behavior schedule	–
Chadwick et al. (133) Uncontrolled study Nonblind study	Group CBT	Yes/not specified	22 CBT no control group	Not specified/SKP and SKA TRP	Eight 1-h weekly sessions over 8 weeks	Significant improvement in mean conviction scores and in the three beliefs	–
Klingberg et al. (150) 2 years follow-up of an RCT Nonblind study	Psychoeducational medication management training (PMT), CBT	Yes/various	191 patients PMT/PMT+ CBT/PMT+ Key person counseling (KC)/PMT+ CBT+KC/TAU	Chronic/age at onset 22.9+ years; 31.3+ years/SKP	10 h of PMT combined with 15 h of CBT and with 15 h of KC/8 months	Not significant difference in BPRS, SANS.	–
Pinto et al. (120) RCT Nonblind study	CBT, SST, ST	Yes/clozapine	20 CBT + SST + clozapine/21 supportive therapy (ST) + clozapine	Not specified/duration of illness: 11.6–11.7++ years/33.9+ CBT, 35.8 ST/SKP	1-h weekly sessions/6 months. Monthly family support	Statistically significant improvement in both groups but no significant difference for negative symptoms.	3
Tarrier et al. (152) One-year follow-up (Tarrier et al. (151), RCT)	CBT, SC	Yes/various	CBT/SC/RC	See Tarrier et al. (137)	See Tarrier et al. (137)	Significant difference in CBT vs. RC for positive symptoms. Nonsignificant improvement for negative symptoms	–
Tarrier et al. (151) RCT Single-blind study	CBT, SC	Yes/typical and atypical antipsychotics	33 intensive CBT+ routine care (RC)/26 SC + RC/28 RC	Heterogeneous/duration of illness 11++ years; 38.6+ years/SKP, SKA, DD	Twenty sessions/10 weeks, 4 booster (B) sessions/4 months; 6 h SC session/10 weeks, 4 B sessions; 20 RC/10 weeks	Significant improvement for positive symptoms in CBT vs. SC and RC, and in SC vs. RC. RC showed slight deterioration. 18 patients achieved 50% improvement in psychotic symptoms: 11 CBT, 4 SC, 3RC	3
Levine et al. (116) Controlled trial Nonblind study	Group CBT	Yes/typical antipsychotics	6 group CBT/6 control group	Chronic; duration of illness: at least 5 years 20–45 years/paranoid SKP	Six 50-min weekly sessions, with a 4-week follow-up	Significant result at PANSS score at 4th and at 6th week and at 4 weeks follow-up in group CBT	–
Kuipers et al. (148) RCT Blind study	Individual CBT	Yes/various Three patients did not assume medication (2 in CBT group,1 in control group)	28 of 60 CBT plus standard care/32 TAU	Heterogeneous/duration of illness: 12.1++ years CBT, 14++ years TAU; 38.5+, CBT, 41.8+ TAU/SKP, SKA, DD	Mean number of 1-h session (flexible)/15 given over 9 months	Significant improvement only in CBT group, who showed a 25% reduction on the BPRS. Three people became worse and one committed suicide	3
Garety et al. (117) Controlled trial Nonblind study	Individual CBT	Yes/not specified	13 CBT/7 TAU (waiting list group)	Chronic/duration of illness 16.5++ years CBT, 10.9++ years TAU; 39,6+ years CBT, 37.6+ years TAU/SKP or SKA	Weekly or fortnightly sessions, up to 22 sessions, with an average of 16 sessions/6 months	Significant improvement in delusions, preoccupation and action at MADS, BPRS in CBT group. No variations in self- esteem, distress, and insight.	–

* According to the entry criteria for an early intervention for psychosis service, defined using PANSS scores of at least 4 on hallucinations or delusions or at least 5 on conceptual disorganization, grandiosity, or suspiciousness, in the context of initial presentation to services with psychotic experiences.

+ Mean age of patients (yrs); ++ Mean duration of illness (yrs).

## Appendix 2

**Table:**

Author/type of study	Psychological intervention	Adjunction/antipsychotic	Number of patients/control group	Stage of illnesses and/or age of patients/diagnosis	Frequency and time sessions/duration of treatment	Results
Hutton, (174) Simple meta-analysis	CBT	See Newton-Howes and Wood, (175)	See Newton-Howes and Wood, (175)	Not specified; 18–65 years/SKP	See Newton-Howes and Wood (175)	Group significant differences at 8–18 months, CBT is more effective
Crawford et al. (176) RCT	Group art therapy	Yes/Various	649/activity groups plus TAU/TAU	417 of 41+ years/17++ years/SKP	Weekly sessions 90 min/12 months	No statistically significant difference
Newton-Howes and Wood (175) Meta-analysis	CBT	Yes/not specified	602/placebo group	Not specified SKA/18–65 years	9 Studies/7–22 weeks/4, 6, 9 months	No significant differences
Lynch et al. (177) Meta-analytical review of well-controlled trial	CBT	Yes/not specified	310 CBT/291 control groups	Acute and chronic adult SKP	From 5 weeks to 9 months	CBT is no better than nonspecific controls and does not reduce relapse rates
Gold et al. (178) Meta-analysis RCT, CCT and pre–post study	Music therapy	Yes/not specified	15 studies (*n* = 691 patients): 8 RCTs, 3 CCTs, 4 uncontrolled studies	Psychotic and nonpsychotic severe mental illness patients	3–51 sessions	Significant effects on general and negative symptoms, with dose effect
Garety et al. (179) RCT	CBT, FI with and without carers	Yes/various	301 (1) 27 without carers TAU or CBT+TA U, (2) 106 with carers TAU, CBT + TAU or FI + TAU	Non affective psychosis/​18–65 years, at least moderate severity for one symptom at PANSS	CBT and FI focusing on relapse prevention, 12–20 1-h sessions/9 months	The CBT and FI had no effects at 12 or 24 months. CBT showed effects on depression at 24 months
Bendall et al. (64) RCT	BF	Yes/various	30 ACE/30 BF	Acute first episode psychosis	Up to 20 sessions, 45 min/14 weeks	BF was comparable to CBT
Talwar et al. (180) RCT	Music therapy	Yes/not specified	33 music therapy + T AU/48 TAU	Inpatients, SKA spectrum	45-min weekly sessions/12 weeks	Significant reduction in PANSS total score
Bechdolf et al. (181) RCT	Group CBT, group PE	Yes/not specified	88/40 CBT/48 PE	One episode of SKP or related disorder, 18–64 years	16 sessions group CBT or 18 sessions group PE/8 weeks	Significant less re-hospitalization at 6 months FU in CBT group
Tarrier et al. (166) RCT	CBT, supportive counseling (SC)	Yes/TAU	101 of 309 CBT + TAU/106 SC + TAU/102 TAU	SKA spectrum or delusional disorder	An 18-month follow-up; 15–20 h plus four “booster” sessions treatment/5 weeks	Improvement at PANSS in both groups for positive and negative symptoms
Shahar et al. (182) Retrospective study	Psychoanalytically oriented treatment	No	29 anaclitic/34 introjective/27 mixed type	Inpatients with psychosis (30%), severe personality disorders (60%) and severe depression (10%)	Treatment including psychoanalytic psychotherapy 4 times a week/15 months	Significant improvement only in the mixed type (anaclitic–introjective) at WAIS, Rorschach, and TAT
Haddock et al. (183) RCT	Individual and family-oriented CBT combined with motivational intervention for substance use problems	Yes/neuroleptics	18 patients and 18 carers. Individual intervention (II) with CBT + motivational intervention combined with FI + TAU/TAU	Schizophrenia spectrum disorder or delusional disorder, 18–35 years and face-to-face contact with a carer for a minimum of 10 h per week.	9 months of motivational intervention with 18-month FU period/II: around 29 sessions. FI: 10–16 sessions use	There was no difference between the two groups for PANSS general or total subscale scores. SFS total scores at 18 months II had significantly superior GAF scores at the 18-month follow-up
Turkington et al. (184) RT	Brief CBT	Yes/not specified antipsychotics	257 of 422 patients CBT/165 standard care	Patients with schizophrenia in secondary care settings	6-h-long sessions over 2–3 months	Improvements at CPRS, IRS, BCQ, and MRS, in overall symptomatology, carer burden, insight into CBT group
Pilling et al. (142) Meta-analysis	Social skill training (SST), cognitive remediation (CR)	Yes/various	SST/CR	Chronic SKP/mean duration of illness: 6 ± 3 years (specified in 7 studies)	1-h session, weekly–fortnightly–monthly	No clear evidence on improvements of SST. No benefits of CR
Lewis et al. (95) RCT Early psychosis in acute phase	CBT	Yes/not specified	101 of 309 patients CBT/106 of supportive counseling/102 routine care	Acute phase of first and second episode within 2 years of treatment/DSM schizophrenia spectrum	15–20 h in 5 weeks plus 1–2 weeks and 1–3 months	PANSS total and positive showed “trend” for the CBT to improve fastest; in 60% hallucination resolution in CBT > SC; TAU > SC
Drury et al. (185) RCT	CBT/recreational activities and support	Yes/various	20 of 40 adjunction CBT/20 with social recreational program	Hospitalized patients suffering from acute episode of nonaffective psychosis	8 h for week treatment for a maximum of 6 months	PAS and PBIQ scores showed no significant variation in positive and negative symptoms
Hogarty et al. (186) Clinical trial	Personal therapy	Yes/minimum effective dose (not specified)	151 randomly assigned to 1) personal therapy, 2) FI PE, 3) mixed therapy 4) ST, 54 patients randomly assigned to 1) personal therapy 2)FI	SKP or SKA disorder patients after hospital discharge	3 years	Personal therapy improves the social adjustment in the 2nd and 3rd years. ST, with or without FI, effective with peak at 12 months. Long-term therapy is more effective
Buchkremer et al. (187) RC intervention study	Psychoeducational medication management training (PMT), cognitive psychotherapy (CP), key-person counseling (KC)	Yes/4,639 ± 680 (mean dose) of chlorpromazine equivalents 40% depot 49% oral 11% combined oral and depot	132 patients/5 group: 32 PMT+ regular leisure-time group (LGT)/ 35 PMT + CP/34 PMT + LG T + KC/33 PMT + CP + KC/57 LGT	SKP 31.3+ years, 22.9+ at onset years, the mean number of hospitalizations: 4.7 ± 3.6, total duration of hospitalization: 56.4 ± 52.5 weeks	PMT: 10 group sessions, the first 5 at weekly interval, then at fortnightly. (6–8 persons per group)	Favorable result in PMT + CP + KC Best results in PMT + CP + KC with 24% lower rehospitalization at 1 year follow-up and 26% at 2 years follow-up

## References

[1] Jones PB Barnes TR Davies L Dunn G Lloyd H Hayhurst KP Randomized controlled trial of the effect on Quality of Life of second-vs first-generation antipsychotic drugs in schizophrenia: Cost Utility of the Latest Antipsychotic Drugs in Schizophrenia Study (CUtLASS 1). Arch Gen Psychiatry (2006) 63(10):1079–87. 10.1001/archpsyc.63.10.1079 17015810

[2] Howes OD McCutcheon R Owen MJ Murray RM The role of genes, stress, and dopamine in the development of schizophrenia. Biol Psychiatry (2017) 81(1):9–20. 10.1016/j.biopsych.2016.07.014 27720198 PMC5675052

[3] Lieberman JA Stroup TS McEvoy JP Swartz MS Rosenheck RA Perkins DO Effectiveness of antipsychotic drugs in patients with chronic schizophrenia. N Eng J Med (2005) 353(12):1209–23. 10.1056/NEJMoa051688 16172203

[4] Elkis H Treatment-resistant schizophrenia. Psychiatr Clin North Am (2007) 30(3):511–33. 10.1016/j.psc.2007.04.001 17720034

[5] Fowler D Garety P Kuipers E Cognitive behaviour therapy for psychosis: theory and practice. Chichester, UK: Wiley (1995).

[6] Miller A McEvoy J Jeste D Marder S Treatment of chronic schizophrenia. In: Textbook of schizophrenia. Washington DC: The American Psychiatric Publishing (2006). p. 365–81.

[7] Miyamoto S Jarskog LF Fleischhacker WW New therapeutic approaches for treatment-resistant schizophrenia: a look to the future. J Psychiatr Res (2014) 58:1–6. 10.1016/j.jpsychires.2014.07.001 25070124

[8] Tarrier N An investigation of residual psychotic symptoms in discharged schizophrenic patients. Br J Clin Psychol (1987) 26(2):141–3. 10.1111/j.2044-8260.1987.tb00740.x 3580649

[9] Andreasen NC Arndt S Alliger R Miller D Flaum M Symptoms of schizophrenia: methods, meanings, and mechanisms. Arch Gen Psychiatry (1995) 52(5):341–51. 10.1001/archpsyc.1995.03950170015003 7726714

[10] Kennedy JL Altar CA Taylor DL Degtiar I Hornberger JC The social and economic burden of treatment-resistant schizophrenia: a systematic literature review. Int Clin Psychopharmacol (2014) 29(2):63–76. 10.1097/YIC.0b013e32836508e6 23995856

[11] de Bartolomeis A Buonaguro EF Latte G Rossi R Marmo F Iasevoli F Immediate-early genes modulation by antipsychotics: translational implications for a putative gateway to drug-induced long-term brain changes. Front Behav Neurosci (2017) 11:240. 10.3389/fnbeh.2017.00240 29321734 PMC5732183

[12] Rund BR Barder HE Evensen J Haahr U Hegelstad WtV Joa I Neurocognition and duration of psychosis: a 10-year follow-up of first-episode patients. Schizophr Bull (2015) 42(1):87–95. 10.1192/bjp.bp.117.201475 26101305 PMC4681546

[13] Kane J Honigfeld G Singer J Meltzer H Clozapine for the treatment-resistant schizophrenic: a double- blind comparison with chlorpromazine. Arch Gen Psychiatry (1988) 45(9):789–96. 10.1001/archpsyc.1988.01800330013001 3046553

[14] Elkis H Buckley PF Treatment-resistant schizophrenia. Psychiatr Clin (2016) 39(2):239–65. 10.1016/j.psc.2007.04.001 27216902

[15] Ajnakina O Horsdal HT Lally J MacCabe JH Murray RM Gasse C Validation of an algorithm-based definition of treatment resistance in patients with schizophrenia. Schizophr Res (2018) 197:294–7. 10.1016/j.schres.2018.02.017 29472163

[16] Leucht S Arbter D Engel R Kissling W Davis J How effective are second-generation antipsychotic drugs? A meta-analysis of placebo-controlled trials. Mol Psychiatry (2009) 14(4):429. 10.1038/sj.mp.4002136 18180760

[17] Remington G Addington D Honer W Ismail Z Raedler T Teehan M Guidelines for the pharmacotherapy of schizophrenia in adults. Can J Psychiatry (2017) 62(9):604–16. 10.1177/0706743717720448 PMC5593252 28703015

[18] Garety PA Fowler D Kuipers E Cognitive-behavioral therapy for medication-resistant symptoms. Schizophr Bull (2000) 26(1):73–86. 10.1093/oxfordjournals.schbul.a033447 10755670

[19] Silverstein SM Bellack AS A scientific agenda for the concept of recovery as it applies to schizophrenia. Clin Psychol Rev (2008) 28(7):1108–24. 10.1016/j.cpr.2008.03.004 18420322

[20] Davidson L Stayner D Haglund KE Phenomenological perspectives on the social functioning of people with schizophrenia. In Mueser KT Tarrier N (Eds.), Handbook of Social Functioning in Schizophrenia. Needham Heights, MA, US: Allyn & Bacon (1998) p. 97–120.

[21] Iasevoli F Giordano S Balletta R Latte G Formato MV Prinzivalli E Treatment resistant schizophrenia is associated with the worst community functioning among severely-ill highly-disabling psychiatric conditions and is the most relevant predictor of poorer achievements in functional milestones. Prog Neuropsychopharmacol Biol Psychiatry (2016) 65:34–48. 10.1016/j.pnpbp.2015.08.010 26320028

[22] de Bartolomeis A Balletta R Giordano S Buonaguro EF Latte G Iasevoli F Differential cognitive performances between schizophrenic responders and non-responders to antipsychotics: correlation with course of the illness, psychopathology, attitude to the treatment and antipsychotics doses. Psychiatry Res (2013) 210(2):387–95. 10.1016/j.psychres.2013.06.042 23910239

[23] Manchanda R Norman RM Malla AK Harricharan R Northcott S Persistent psychoses in first episode patients. Schizophr Res (2005) 80(1):113–6. 10.1016/j.schres.2005.08.005 16171975

[24] Wiersma D Nienhuis FJ Slooff CJ Giel R Natural course of schizophrenic disorders: a 15-year followup of a Dutch incidence cohort. Schizophr Bull (1998) 24(1):75–85. 10.1093/oxfordjournals.schbul.a033315 9502547

[25] Meltzer H Kostacoglu A Treatment-resistant schizophrenia. In: Comprehensive care of schizophrenia: a textbook of clinical management. London: Martin Dunitz (2001). p. 181–203.

[26] Lally J Ajnakina O Di Forti M Trotta A Demjaha A Kolliakou A Two distinct patterns of treatment resistance: clinical predictors of treatment resistance in first-episode schizophrenia spectrum psychoses. Psychol Med (2016) 46(15):3231–40. 10.1017/S0033291716002014 27605254

[27] Lee J Takeuchi H Fervaha G Sin GL Foussias G Agid O Subtyping schizophrenia by treatment response: antipsychotic development and the central role of positive symptoms. Focus (2016) 14(3):396–402. 10.1176/appi.focus.140306 PMC6526794 31997961

[28] Sheitman B Lieberman J The natural history and pathophysiology of treatment resistant schizophrenia. J Psychiatr Res (1998) 32(3–4):143–50. 10.1016/S0022-3956(97)00052-6 9793867

[29] de Bartolomeis A Sarappa C Magara S Iasevoli F Targeting glutamate system for novel antipsychotic approaches: relevance for residual psychotic symptoms and treatment resistant schizophrenia. Eur J Pharmacol (2012) 682(1–3):1–11. 10.1016/j.ejphar.2012.02.033 22387855

[30] Demjaha A Egerton A Murray RM Kapur S Howes OD Stone JM Antipsychotic treatment resistance in schizophrenia associated with elevated glutamate levels but normal dopamine function. Biol Psychiatry (2014) 75(5):e11–e3. 10.1016/j.biopsych.2013.06.011 23890739

[31] Buchanan RW Clozapine: efficacy and safety. Schizophr Bull (1995) 21(4):579–91. 10.1093/schbul/21.4.579 8749886

[32] Kane JM Correll CU Pharmacologic treatment of schizophrenia. Dialogues Clin Neurosci (2010) 12(3):345–57. 10.31887/DCNS.2010.12.3/jkane PMC3085113 20954430

[33] Young CR Longhurst JG Bowers MB Jr. Mazure CM The expanding indications for clozapine. Exp Clin Psychopharmacol (1997) 5(3):216. 10.1037/1064-1297.5.3.216 9260069

[34] Lewis S Davies L Jones P Barnes T Murray R Kerwin R Randomised controlled trials of conventional antipsychotic versus new atypical drugs, and new atypical drugs versus clozapine, in people with schizophrenia responding poorly to, or intolerant of, current drug treatment. Health Technol Assess (2006) 10(17):iii–iv, ix–xi, 1–165. 10.3310/hta10170 16707074

[35] Kane JM Correll CU The role of clozapine in treatment-resistant schizophrenia. JAMA Psychiatry (2016) 73(3):187–8. 10.1001/jamapsychiatry.2015.2966 26841681

[36] Samara MT Dold M Gianatsi M Nikolakopoulou A Helfer B Salanti G Efficacy, acceptability, and tolerability of antipsychotics in treatment-resistant schizophrenia: a network meta-analysis. JAMA Psychiatry (2016) 73(3):199–210. 10.1001/jamapsychiatry.2015.2955 26842482

[37] Bruno A Zoccali RA Abenavoli E Pandolfo G Scimeca G Spina E Augmentation of clozapine with agomelatine in partial-responder schizophrenia: a 16-week, open-label, uncontrolled pilot study. J Clin Psychopharmacol (2014) 34(4):491–4. 10.1097/JCP.0000000000000157 24911437

[38] Bruno A Pandolfo G Romeo VM Mallamace D D’arrigo C Spina E Duloxetine as adjunctive treatment to clozapine in patients with schizophrenia: a randomized, placebo-controlled trial. Int Clin Psychopharmacol (2011) 26(6):303–10. 10.1097/YIC.0b013e32834bbc0d 21934625

[39] Muscatello MRA Bruno A De Fazio P Segura-Garcia C Pandolfo G Zoccali R Augmentation strategies in partial responder and/or treatment-resistant schizophrenia patients treated with clozapine. Expert Opin Pharmacother (2014) 15(16):2329–45. 10.1517/14656566.2014.956082 25284216

[40] Buckley P Miller A Olsen J Garver D Miller DD Csernansky J When symptoms persist: clozapine augmentation strategies. Schizophr Bull (2001) 27(4):615–28. 10.1093/oxfordjournals.schbul.a006901 11824488

[41] Zink M Augmentation of olanzapine in treatment-resistant schizophrenia. J Psychiatry Neurosci (2005) 30(6):409–15. PMC1277023 16327874

[42] Lindenmayer J-P Treatment refractory schizophrenia. Psychiatr Quart (2000) 71(4):373–84. 10.1023/A:1004640408501 11025914

[43] Addington J Van Mastrigt S Addington D Duration of untreated psychosis: impact on 2-year outcome. Psychol Med (2004) 34(2):277–84. 10.1017/S0033291703001156 14982133

[44] Barnes TR Leeson VC Mutsatsa SH Watt HC Hutton SB Joyce EM Duration of untreated psychosis and social function: 1-year follow-up study of first-episode schizophrenia. Br J Psychiatry (2008) 193(3):203–9. 10.1192/bjp.bp.108.049718 PMC2576506 18757977

[45] Carbon M Correll CU Clinical predictors of therapeutic response to antipsychotics in schizophrenia. Dialogues Clin Neurosci (2014) 16(4):505–24. 10.31887/DCNS.2014.16.4/mcarbon PMC4336920 25733955

[46] Larsen TK McGlashan TH Moe LC First-episode schizophrenia: I. Schizophr Bull (1996) 22(2):241–56. 10.1093/schbul/22.2.241 8782284

[47] Meltzer HY Rabinowitz J Lee MA Cola PA Ranjan R Findling RL Age at onset and gender of schizophrenic patients in relation to neuroleptic resistance. Am J Psychiatry (1997) 154(4):475–82. 10.1176/ajp.154.4.475 9090333

[48] Singh SP Merino C Treatment of first-episode and prodromal signs. Psychiatry (2008) 7(11):467–71. 10.1016/j.mppsy.2008.10.001

[49] Caspi A Davidson M Tamminga CA Treatment-refractory schizophrenia. Dialogues Clin Neurosci (2004) 6(1):61–70. 22034144 10.31887/DCNS.2004.6.1/acaspi PMC3181784

[50] Johnstone E Owens D Bydder G Colter N Crow T Frith C The spectrum of structural brain changes in schizophrenia: age of onset as a predictor of cognitive and clinical impairments and their cerebral correlates. Psychol Med (1989) 19(1):91–103. 10.1017/S0033291700011053 2727213

[51] Kolakowska T Williams A Ardern M Reveley M Jambor K Gelder M Schizophrenia with good and poor outcome. Br J Psychiatry (1985) 146(3):229–39. 10.1192/bjp.146.3.229 2859067

[52] Nakajima S Takeuchi H Plitman E Fervaha G Gerretsen P Caravaggio F Neuroimaging findings in treatment-resistant schizophrenia: a systematic review: lack of neuroimaging correlates of treatment-resistant schizophrenia. Schizophr Res (2015) 164(1–3):164–75. 10.1016/j.schres.2015.01.043 PMC4409508 25684554

[53] Quarantelli M Palladino O Prinster A Schiavone V Carotenuto B Brunetti A Patients with poor response to antipsychotics have a more severe pattern of frontal atrophy: a voxel-based morphometry study of treatment resistance in schizophrenia. BioMed Res Int (2014) 2014:1–9. 10.1155/2014/325052 PMC4135095 25157354

[54] Rosenbaum B Harder S Knudsen P Køster A Lindhardt A Lajer M Supportive psychodynamic psychotherapy versus treatment as usual for first-episode psychosis: two-year outcome. Psychiatry (2012) 75(4):331–41. 10.1521/psyc.2012.75.4.331 23244011

[55] Fonagy P The effectiveness of psychodynamic psychotherapies: an update. World Psychiatry (2015) 14(2):137–50. 10.1002/wps.20235 PMC4471961 26043322

[56] Budd R Hughes I The Dodo Bird Verdict—controversial, inevitable and important: a commentary on 30 years of meta-analyses. Clin Psychol Psychother (2009) 16(6):510–22. 10.1002/cpp.648 19728292

[57] Gottdiener WH Haslam N The benefits of individual psychotherapy for people diagnosed with schizophrenia: a meta-analytic review. Ethical Hum Sci Serv (2002) 4(3):163–87. 10.1891/1523-150X.4.3.163

[58] Malmberg L Fenton M Rathbone J Individual psychodynamic psychotherapy and psychoanalysis for schizophrenia and severe mental illness. Cochrane Database Syst Rev (2001) 3:CD001360. 10.1002/14651858.CD001360 PMC4171459 11686988

[59] Paley G Shapiro DA Lessons from psychotherapy research for psychological interventions for people with schizophrenia. Psychol Psychother Theor Res Pract (2002) 75(1):5–17. 10.1348/147608302169517 12006196

[60] Castelnuovo G Empirically supported treatments in psychotherapy: towards an evidence-based or evidence-biased psychology in clinical settings? Front Psychol (2010) 1:27. 10.3389/fpsyg.2010.00027 21833197 PMC3153746

[61] Kazdin AE Evidence-based treatment and usual care: cautions and qualifications. JAMA Psychiatry (2013) 70(7):666–7. 10.1001/jamapsychiatry.2013.2112 23754003

[62] Weisz JR Kuppens S Eckshtain D Ugueto AM Hawley KM Jensen-Doss A Performance of evidence- based youth psychotherapies compared with usual clinical care: a multilevel meta-analysis. JAMA Psychiatry (2013) 70(7):750–61. 10.1001/jamapsychiatry.2013.1176 PMC3848075 23754332

[63] Bendall S Killackey E Jackson H Gleeson J Befriending manual. Melbourne: ORYGEN Research Centre, University of Melbourne (2003).

[64] Bendall S Jackson HJ Killackey E Allott K Johnson T Harrigan S The credibility and acceptability of befriending as a control therapy in a randomized controlled trial of cognitive behaviour therapy for acute first episode psychosis. Behav Cogn Psychother (2006) 34(3):277–91. 10.1017/S1352465806002815

[65] Beck AT Successful outpatient psychotherapy of a chronic schizophrenic with a delusion based on borrowed guilt. Psychiatry (1952) 15(3):305–12. 10.1080/00332747.1952.11022883 12983446

[66] Shapiro MB Ravenette A A preliminary experiment on paranoid delusions. J Ment Science (1959) 105(439):295–312. 10.1192/bjp.105.439.295 13665292

[67] Health NCCfM Schizophrenia: core interventions in the treatment and management of schizophrenia in primary and secondary care (update). Leicester, UK: British Psychological Society (2009). 20704054

[68] Grant N Lawrence M Preti A Wykes T Cella M Social cognition interventions for people with schizophrenia: a systematic review focussing on methodological quality and intervention modality. Clin Psychol Rev (2017) 56:55–64. 10.1016/j.cpr.2017.06.001 28688282

[69] Hazell CM Hayward M Cavanagh K Strauss C A systematic review and meta-analysis of low intensity CBT for psychosis. Clin Psychol Rev (2016) 45:183–92. 10.1016/j.cpr.2016.03.004 27048980

[70] Tarrier N Haddock G Barrowclough C Wykes T Are all psychological treatments for psychosis equal? The need for CBT in the treatment of psychosis and not for psychodynamic psychotherapy. Psychol Psychother Theor Res Pract (2002) 75(4):365–74. 10.1348/147608302321151871 12626130

[71] Mueser KT Berenbaum H Psychodynamic treatment of schizophrenia: is there a future? Psychol Med (1990) 20(2):253–62. 10.1017/S003329170001758X 2192381

[72] Cicchetti D Doyle C Child maltreatment, attachment and psychopathology: mediating relations. World Psychiatry (2016) 15(2):89–90. 10.1002/wps.20337 27265688 PMC4911777

[73] Cristofaro SL Cleary SD Wan CR Broussard B Chapman C Haggard PJ Measuring trauma and stressful events in childhood and adolescence among patients with first-episode psychosis: initial factor structure, reliability, and validity of the Trauma Experiences Checklist. Psychiatry Res (2013) 210(2):618–25. 10.1016/j.psychres.2013.06.015 PMC3816125 23850437

[74] Egerton A Valmaggia LR Howes OD Day F Chaddock CA Allen P Adversity in childhood linked to elevated striatal dopamine function in adulthood. Schizophr Res (2016) 176(2–3):171–6. 10.1016/j.schres.2016.06.005 PMC5147458 27344984

[75] Fisher HL Jones PB Fearon P Craig TK Dazzan P Morgan K The varying impact of type, timing and frequency of exposure to childhood adversity on its association with adult psychotic disorder. Psychol Med (2010) 40(12):1967–78. 10.1017/S0033291710000231 PMC3272393 20178679

[76] Janssen I Krabbendam L Bak M Hanssen M Vollebergh W de Graaf R Childhood abuse as a risk factor for psychotic experiences. Acta Psychiatr Scand (2004) 109(1):38–45. 10.1046/j.0001-690X.2003.00217.x 14674957

[77] Morgan C Gayer-Anderson C Childhood adversities and psychosis: evidence, challenges, implications. World Psychiatry (2016) 15(2):93–102. 10.1002/wps.20330 27265690 PMC4911761

[78] Murray RM Mehta M Di Forti M Different dopaminergic abnormalities underlie cannabis dependence and cannabis-induced psychosis. Biol Psychiatry (2014) 75(6):430–1. 10.1016/j.biopsych.2014.01.011 24560431

[79] Varese F Smeets F Drukker M Lieverse R Lataster T Viechtbauer W Childhood adversities increase the risk of psychosis: a meta-analysis of patient-control, prospective-and cross-sectional cohort studies. Schizophr Bull (2012) 38(4):661–71. 10.1093/schbul/sbs050 PMC3406538 22461484

[80] Galatzer-Levy IR Huang SH Bonanno GA Trajectories of resilience and dysfunction following potential trauma: a review and statistical evaluation. Clin Psychol Rev (2018) 63:41–55. 10.1016/j.cpr.2018.05.008 29902711

[81] Maccari S Polese D Reynaert M-L Amici T Morley-Fletcher S Fagioli F Early-life experiences and the development of adult diseases with a focus on mental illness: the human birth theory. Neuroscience (2017) 342:232–51. 10.1016/j.neuroscience.2016.05.042 27235745

[82] Priebe S McCabe R The therapeutic relationship in psychiatric settings. Acta Psychiatr Scand (2006) 113:69–72. 10.1111/j.1600-0447.2005.00721.x 16445486

[83] Priebe S Mccabe R Therapeutic relationships in psychiatry: the basis of therapy or therapy in itself? Int Rev Psychiatry (2008) 20(6):521–6. 10.1080/09540260802565257 19085408

[84] Priebe S Richardson M Cooney M Adedeji O McCabe R Does the therapeutic relationship predict outcomes of psychiatric treatment in patients with psychosis? A systematic review. Psychother Psychosom (2011) 80(2):70–7. 10.1159/000320976 21196804

[85] Harper Romeo K Meyer PS Johnson D Penn DL An investigation of the relationship between therapist characteristics and alliance in group therapy for individuals with treatment-resistant auditory hallucinations. J Ment Health (2014) 23(4):166–70. 10.3109/09638237.2013.869568 25054367

[86] Calton T Ferriter M Huband N Spandler H A systematic review of the Soteria paradigm for the treatment of people diagnosed with schizophrenia. Schizophr Bull (2007) 34(1):181–92. 10.1093/schbul/sbm047 PMC2632384 17573357

[87] Ganasen K Ipser J Stein D Augmentation of cognitive behavioral therapy with pharmacotherapy. Psychiatr Clin North Am (2010) 33(3):687–99. 10.1016/j.psc.2010.04.008. 20599140

[88] Lauriello J Lenroot R Bustillo JR Maximizing the synergy between pharmacotherapy and psychosocial therapies for schizophrenia. Psychiatr Clin North Am (2003) 26(1):191–211. 10.1016/S0193-953X(02)00017-5 12683266

[89] Rathod S Kingdon D Weiden P Turkington D Cognitive-behavioral therapy for medication-resistant schizophrenia: a review. J Psychiatr Pract (2008) 14(1):22–33. 10.1097/01.pra.0000308492.93003.db 18212600

[90] Plakun E Treatment resistance and psychodynamic psychiatry: concepts psychiatry needs from psychoanalysis. Psychodyn Psychiatry (2012) 40(2):183–209. 10.1521/pdps.2012.40.2.183 23006116

[91] Harder S Koester A Valbak K Rosenbaum B Five-year follow-up of supportive psychodynamic psychotherapy in first-episode psychosis: long-term outcome in social functioning. Psychiatry (2014) 77(2):155–68. 10.1521/psyc.2014.77.2.155 24865198

[92] Craig TK Garety P Power P Rahaman N Colbert S Fornells-Ambrojo M The Lambeth Early Onset (LEO) Team: randomised controlled trial of the effectiveness of specialised care for early psychosis. Bmj (2004) 329(7474):1067. 10.1136/bmj.38246.594873.7C 15485934 PMC526115

[93] Birchwood M Michail M Meaden A Tarrier N Lewis S Wykes T Cognitive behaviour therapy to prevent harmful compliance with command hallucinations (COMMAND): a randomised controlled trial. Lancet Psychiatry (2014) 1(1):23–33. 10.1016/S2215-0366(14)70247-0 26360400

[94] Haddock G Tarrier N Morrison A Hopkins R Drake R Lewis S A pilot study evaluating the effectiveness of individual inpatient cognitive-behavioural therapy in early psychosis. Soc Psychiatry Psychiatr Epidemiol (1999) 34(5):254–8. 10.1007/s001270050141 10396167

[95] Lewis S Tarrier N Haddock G Bentall R Kinderman P Kingdon D Randomised controlled trial of cognitive-behavioural therapy in early schizophrenia: acute-phase outcomes. Br J Psychiatry (2002) 181(S43):s91–s7. 10.1192/bjp.181.43.s91 12271807

[96] Remington G Kapur S Zipursky RB Pharmacotherapy of first-episode schizophrenia. Br J Psychiatry (1998) 172(S33):66–70. 10.1192/S0007125000297687 9764129

[97] Dorph-Petersen K-A Pierri JN Perel JM Sun Z Sampson AR Lewis DA The influence of chronic exposure to antipsychotic medications on brain size before and after tissue fixation: a comparison of haloperidol and olanzapine in macaque monkeys. Neuropsychopharmacology (2005) 30(9):1649. 10.1038/sj.npp.1300710 15756305

[98] Torres US Duran FL Schaufelberger MS Crippa JA Louzã MR Sallet PC Patterns of regional gray matter loss at different stages of schizophrenia: a multisite, cross-sectional VBM study in first-episode and chronic illness. Neuroimage Clin (2016) 12:1–15. 10.1016/j.nicl.2016.06.002 27354958 PMC4910144

[99] Vita A De Peri L Deste G Barlati S Sacchetti E The effect of antipsychotic treatment on cortical gray matter changes in schizophrenia: does the class matter? A meta-analysis and meta-regression of longitudinal magnetic resonance imaging studies. Biol Psychiatry (2015) 78(6):403–12. 10.1016/j.biopsych.2015.02.008 25802081

[100] Chouinard G Samaha A-N Chouinard V-A Peretti C-S Kanahara N Takase M Antipsychotic- induced dopamine supersensitivity psychosis: pharmacology, criteria, and therapy. Psychother Psychosom (2017) 86(4):189–219. 10.1159/000477313 28647739

[101] Chouinard G Chouinard V-A Atypical antipsychotics: CATIE study, drug-induced movement disorder and resulting iatrogenic psychiatric-like symptoms, supersensitivity rebound psychosis and withdrawal discontinuation syndromes. Psychother Psychosom (2008) 77(2):69–77. 10.1159/000112883 18230939

[102] Samaha A-N Seeman P Stewart J Rajabi H Kapur S “Breakthrough” dopamine supersensitivity during ongoing antipsychotic treatment leads to treatment failure over time. J Neurosci (2007) 27(11):2979–86. 10.1523/JNEUROSCI.5416-06.2007 PMC6672560 17360921

[103] Edwards J Maude D Herrmann-Doig T Wong L Cocks J Burnett P Rehab rounds: a service response to prolonged recovery in early psychosis. Psychiatr Serv (2002) 53(9):1067–9. 10.1176/appi.ps.53.9.1067 12221301

[104] Morrison AP French P Walford L Lewis SW Kilcommons A Green J Cognitive therapy for the prevention of psychosis in people at ultra-high risk: randomised controlled trial. Br J Psychiatry (2004) 185(4):291–7. 10.1192/bjp.185.4.291 15458988

[105] Kaur T Cadenhead KS Treatment implications of the schizophrenia prodrome. In: Behavioral Neurobiology of Schizophrenia and Its Treatment. Berlin Heidelberg, Germany: Springer (2010). p. 97–121. 10.1007/7854_2010_56 PMC3136161 21312398

[106] McGorry PD Nelson B Amminger GP Bechdolf A Francey SM Berger G Intervention in individuals at ultra-high risk for psychosis: a review and future directions. J Clin Psychiatry (2009) 70(9):1206–12. 10.4088/JCP.08r04472 19573499

[107] McGorry PD Hickie IB Yung AR Pantelis C Jackson HJ Clinical staging of psychiatric disorders: a heuristic framework for choosing earlier, safer and more effective interventions. Austr N Z J psychiatry (2006) 40(8):616–22. 10.1080/j.1440-1614.2006.01860.x 16866756

[108] National Collaborating Centre for Mental Health (UK) Schizophrenia: core interventions in the treatment and management of schizophrenia in primary and secondary care (Update). Leicester, UK: British Psychological Society (2009). Available from http://www.ncbi.nlm.nih.gov/books/NBK11681/PubMed. 20704054

[109] Lehman AF Kreyenbuhl J Buchanan RW Dickerson FB Dixon LB Goldberg R The schizophrenia patient outcomes research team (PORT): updated treatment recommendations 2003. Schizophr Bull (2004) 30(2):193–217. 10.1093/oxfordjournals.schbul.a007071 15279040

[110] Moher D Liberati A Tetzlaff J Altman DG Group P Preferred reporting items for systematic reviews and meta-analyses: the PRISMA statement. PLoS Med (2009) 6(7):e1000097. 10.1371/journal.pmed.1000097 19621072 PMC2707599

[111] McGauran N Wieseler B Kreis J Schuler YB Kolsch H Kaiser T Reporting bias in medical research - a narrative review. Trials (2010) 11:37. 10.1186/1745-6215-11-37 20388211 PMC2867979

[112] Ross K Freeman D Dunn G Garety P A randomized experimental investigation of reasoning training for people with delusions. Schizophr Bull (2009) 37(2):324–33. 10.1093/schbul/sbn165 PMC3044626 19520745

[113] Cather C Penn D Otto MW Yovel I Mueser KT Goff DC A pilot study of functional Cognitive Behavioral Therapy (fCBT) for schizophrenia. Schizophr Res (2005) 74(2–3):201–9. 10.1016/j.schres.2004.05.002 15722000

[114] Randal P Simpson AI Laidlaw T Can recovery-focused multimodal psychotherapy facilitate symptom and function improvement in people with treatment-resistant psychotic illness? A comparison study. Austr N Z J psychiatry (2003) 37(6):720–7. 10.1080/j.1440-1614.2003.01261.x 14636388

[115] Durham RC Guthrie M Morton RV Reid DA Treliving LR Fowler D Tayside–Fife clinical trial of cognitive–behavioural therapy for medication-resistant psychotic symptoms: results to 3-month follow-up. Br J Psychiatry (2003) 182(4):303–11. 10.1192/bjp.182.4.303 12668405

[116] Levine J Barak Y Granek I Cognitive group therapy for paranoid schizophrenics: applying cognitive dissonance. J Cogn Psychother (1998) 12(1):3. 10.1891/0889-8391.12.1.3

[117] Garety P Kuipers L Fowler D Chamberlain F Dunn G Cognitive behavioural therapy for drug-resistant psychosis. Br J Med Psychol (1994) 67(3):259–71. 10.1111/j.2044-8341.1994.tb01795.x 7803318

[118] Wells G Shea B O’Connell D Peterson J Welch V Losos M The Newcastle-Ottawa Scale (NOS) for assessing the quality of nonrandomised studies in meta-analyses. (2013). [Accessed 2019, April 8, on http://www.ohri.ca/programs/clinical_epidemiology/oxford.asp].

[119] Jadad AR Moore RA Carroll D Jenkinson C Reynolds DJM Gavaghan DJ Assessing the quality of reports of randomized clinical trials: is blinding necessary? Control Clin Trials (1996) 17(1):1–12. 10.1016/0197-2456(95)00134-4 8721797

[120] Pinto A Pia SL Mennella R Giorgio D DeSimone L Rehab rounds: cognitive-behavioral therapy and clozapine for clients with treatment-refractory schizophrenia. Psychiatr Serv (1999) 50(7):901–4. 10.1176/ps.50.7.901 10402608

[121] Valmaggia LR Van Der Gaag M Tarrier N Pijnenborg M Slooff CJ Cognitive–behavioural therapy for refractory psychotic symptoms of schizophrenia resistant to atypical antipsychotic medication: randomised controlled trial. Br J Psychiatry (2005) 186(4):324–30. 10.1192/bjp.186.4.324 15802690

[122] Shawyer F Farhall J Mackinnon A Trauer T Sims E Ratcliff K A randomised controlled trial of acceptance-based cognitive behavioural therapy for command hallucinations in psychotic disorders. Behav Res Ther (2012) 50(2):110–21. 10.1016/j.brat.2011.11.007 22186135

[123] Jacobsen P Morris E Johns L Hodkinson K Mindfulness groups for psychosis; key issues for implementation on an inpatient unit. Behav Cogn Psychother (2011) 39(3):349–53. 10.1017/S1352465810000639 21092359

[124] Temple S Ho B-C Cognitive therapy for persistent psychosis in schizophrenia: a case-controlled clinical trial. Schizophr Res (2005) 74(2–3):195–9. 10.1016/j.schres.2004.05.013 15721999

[125] Rector NA Seeman MV Segal ZV Cognitive therapy for schizophrenia: a preliminary randomized controlled trial. Schizophr Res (2003) 63(1–2):1–11. 10.1016/S0920-9964(02)00308-0 12892853

[126] Waller H Freeman D Jolley S Dunn G Garety P Targeting reasoning biases in delusions: a pilot study of the Maudsley Review Training Programme for individuals with persistent, high conviction delusions. J Behav Ther Exp Psychiatry (2011) 42(3):414–21. 10.1016/j.jbtep.2011.03.001 PMC3145959 21481815

[127] Hutton P Morrison AP Wardle M Wells A Metacognitive therapy in treatment-resistant psychosis: a multiple-baseline study. Behav Cogn Psychother (2014) 42(2):166–85. 10.1017/S1352465812001026 23286558

[128] Trower P Birchwood M Meaden A Byrne S Nelson A Ross K Cognitive therapy for command hallucinations: randomised controlled trial. Br J Psychiatry (2004) 184(4):312–20. 10.1192/bjp.184.4.312 15056575

[129] Mandić-Gajić G Group art therapy as adjunct therapy for the treatment of schizophrenic patients in day hospital. Vojnosanitet Pregl (2013) 70(11):1065–9. 10.2298/VSP1311065M 24397206

[130] Buchain PC Vizzotto ADB Henna Neto J Elkis H Randomized controlled trial of occupational therapy in patients with treatment-resistant schizophrenia. Rev Bras Psiquiatr (2003) 25(1):26–30. 10.1590/S1516-44462003000100006 12975676

[131] Davenport S Hobson R Margison F Treatment development in psychodynamic interpersonal psychotherapy (Hobson’s ‘Conversational Model’) for chronic treatment resistant schizophrenia: two single case studies. Br J Psychother (2000) 16(3):287–302. 10.1111/j.1752-0118.2000.tb00520.x

[132] Barrowclough C Haddock G Lobban F Jones S Siddle R Roberts C Group cognitive-behavioural therapy for schizophrenia: randomised controlled trial. Br J Psychiatry (2006) 189(6):527–32. 10.1192/bjp.bp.106.021386 17139037

[133] Chadwick P Sambrooke S Rasch S Davies E Challenging the omnipotence of voices: group cognitive behavior therapy for voices. Behav Res Ther (2000) 38(10):993–1003. 10.1016/S0005-7967(99)00126-6 11004738

[134] Johnson DP Penn DL Bauer DJ Meyer P Evans E Predictors of the therapeutic alliance in group therapy for individuals with treatment-resistant auditory hallucinations. Br J Clin Psychol (2008) 47(2):171–84. 10.1348/014466507X241604 17900393

[135] Penn DL Meyer PS Evans E Wirth RJ Cai K Burchinal M A randomized controlled trial of group cognitive-behavioral therapy vs. Schizophr Res (2009) 109(1–3):52–9. 10.1016/j.schres.2008.12.009 19176275

[136] Pinkham AE Gloege AT Flanagan S Penn DL Group cognitive-behavioral therapy for auditory hallucinations: a pilot study. Cogn Behav Pract (2004) 11(1):93–8. 10.1016/S1077-7229(04)80011-7

[137] Wykes T Hayward P Thomas N Green N Surguladze S Fannon D What are the effects of group cognitive behaviour therapy for voices? A randomised control trial. Schizophr Res (2005) 77(2–3):201–10. 10.1016/j.schres.2005.03.013 15885983

[138] Pilling S Bebbington P Kuipers E Garety P Geddes J Orbach G Psychological treatments in schizophrenia: I. Psychol Med (2002) 32(5):763–82. 10.1017/S0033291702005895 12171372

[139] de Paiva Barretto EM Kayo M Avrichir BS Sa AR Camargo MDGM Napolitano IC A preliminary controlled trial of cognitive behavioral therapy in clozapine-resistant schizophrenia. J Nerv Ment Dis (2009) 197(11):865–8. 10.1097/NMD.0b013e3181be7422 19996727

[140] Sensky T Turkington D Kingdon D Scott JL Scott J Siddle R A randomized controlled trial of cognitive-behavioral therapy for persistent symptoms in schizophrenia resistant to medication. Arch Gen Psychiatry (2000) 57(2):165–72. 10.1001/archpsyc.57.2.165 10665619

[141] Brabban A Tai S Turkington D Predictors of outcome in brief cognitive behavior therapy for schizophrenia. Schizophr Bull (2009) 35(5):859–64. 10.1093/schbul/sbp065 PMC2728819 19571248

[142] Pilling S Bebbington P Kuipers E Garety P Geddes J Martindale B Psychological treatments in schizophrenia: II. Psychol Med (2002) 32(5):783–91. 10.1017/S0033291702005640 12171373

[143] Peters E Landau S McCrone P Cooke M Fisher P Steel C A randomised controlled trial of cognitive behaviour therapy for psychosis in a routine clinical service. Acta Psychiatr Scand (2010) 122(4):302–18. 10.1111/j.1600-0447.2010.01572.x 20491720

[144] Erickson DH Cognitive-behaviour therapy for medication-resistant positive symptoms in early psychosis: a case series. Early Interv Psychiatry (2010) 4(3):251–6. 10.1111/j.1751-7893.2010.00184.x 20712731

[145] Burns AM Erickson DH Brenner CA Cognitive-behavioral therapy for medication-resistant psychosis: a meta-analytic review. Psychiatr Serv (2014) 65(7):874–80. 10.1176/appi.ps.201300213 24686725

[146] Jones C Hacker D Cormac I Meaden A Irving CB Cognitive behavioural therapy versus other psychosocial treatments for schizophrenia. Cochrane Database Syst Rev (2012) 4:CD008712. 10.1002/14651858.CD008712.pub2 PMC4163968 22513966

[147] Wiersma D Jenner J de Willige G Spakman M Nienhuis F Cognitive behaviour therapy with coping training for persistent auditory hallucinations in schizophrenia: a naturalistic follow-up study of the durability of effects. Acta Psychiatr Scand (2001) 103(5):393–9. 10.1034/j.1600-0447.2001.00213.x 11380310

[148] Kuipers E Garety P Fowler D Dunn G Bebbington P Freeman D London–East Anglia randomised controlled trial of cognitive–behavioural therapy for psychosis: I: Effects of the treatment phase. Br J Psychiatry (1997) 171(4):319–27. 10.1192/bjp.171.4.319 9373419

[149] Klingberg S Herrlich J Wiedemann G Wölwer W Meisner C Engel C Adverse effects of cognitive behavioral therapy and cognitive remediation in schizophrenia: results of the treatment of negative symptoms study. J Nerv Ment Dis (2012) 200(7):569–76. 10.1097/NMD.0b013e31825bfa1d 22759932

[150] Klingberg S Buchkremer G Holle R Mönking HS Hornung WP Differential therapy effects of psychoeducational psychotherapy for schizophrenic patients–results of a 2-year follow-up. Eur Arch Psychiatry Clin Neurosci (1999) 249(2):66–72. 10.1007/s004060050068 10369152

[151] Tarrier N Yusupoff L Kinney C McCarthy E Gledhill A Haddock G Randomised controlled trial of intensive cognitive behaviour therapy for patients with chronic schizophrenia. Bmj (1998) 317(7154):303–7. 10.1136/bmj.317.7154.303 PMC28621 9685273

[152] Tarrier N Witttkowskj A Kinney C McCarthy E Morris J Humphreys L Durability of the effects of cognitive–behavioural therapy in the treatment of chronic schizophrenia: 12-month follow-up. Br J Psychiatry (1999) 174(6):500–4. 10.1192/bjp.174.6.500 10616627

[153] Tarrier N Kinney C McCarthy E Humphreys L Wittkowski A Morris J Two–year follow–up of cognitive–behavioral therapy and supportive counseling in the treatment of persistent symptoms in chronic schizophrenia. J Consult Clin Psychol (2000) 68(5):917. 10.1037/0022-006X.68.5.917 11068978

[154] Lutgens D Gariepy G Malla A Psychological and psychosocial interventions for negative symptoms in psychosis: systematic review and meta-analysis. Br J Psychiatry (2017) 210(5):324–32. 10.1192/bjp.bp.116.197103 28302699

[155] Bighelli I Salanti G Huhn M Schneider-Thoma J Krause M Reitmeir C Psychological interventions to reduce positive symptoms in schizophrenia: systematic review and network meta-analysis. World Psychiatry (2018) 17(3):316–29. 10.1002/wps.20577 PMC6127754 30192101

[156] Koren D Scheyer R Reznik N Adres M Apter A Parnas J Basic self-disturbance, neurocognition and metacognition: a pilot study among help-seeking adolescents with and without attenuated psychosis. Early Interv Psychiatry (2017). 10.1111/eip.12500 29052951

[157] Parnas J Sass LA Self, solipsism, and schizophrenic delusions. Philos Psychiatr Psychol (2001) 8(2):101–20. 10.1353/ppp.2001.0014

[158] Beck AT Rector NA Cognitive therapy of schizophrenia: a new therapy for the new millennium. Am J Psychother (2000) 54(3):291–300. 10.1176/appi.psychotherapy.2000.54.3.291 11008627

[159] Nelson B Yung AR Bechdolf A McGorry PD The phenomenological critique and self-disturbance: implications for ultra-high risk (“prodrome”) research. Schizophr Bull (2007) 34(2):381–92. 10.1093/schbul/sbm094 PMC2632406 17702990

[160] Mizrahi R Addington J Rusjan PM Suridjan I Ng A Boileau I Increased stress-induced dopamine release in psychosis. Biol Psychiatry (2012) 71(6):561–7. 10.1016/j.biopsych.2011.10.009 22133268

[161] Karlsson H Hirvonen J Kajander J Markkula J Rasi-Hakala H Salminen J Psychotherapy increases brain serotonin 5-HT 1A receptors in patients with major depressive disorder. Psychol Med (2010) 40(3):523–8. 10.1017/S0033291709991607 19903365

[162] Lai C Daini S Calcagni ML Bruno I De Risio S Neural correlates of psychodynamic psychotherapy in borderline disorders–a pilot investigation. Psychother Psychosom (2007) 76(6):403–5. 10.1159/000107572 17917480

[163] Lehto SM Tolmunen T Joensuu M Saarinen PI Valkonen-Korhonen M Vanninen R Changes in midbrain serotonin transporter availability in atypically depressed subjects after one year of psychotherapy. Prog Neuropsychopharmacol Biol Psychiatry (2008) 32(1):229–37. 10.1016/j.pnpbp.2007.08.013 17884269

[164] Paquette V Lévesque J Mensour B Leroux J-M Beaudoin G Bourgouin P “Change the mind and you change the brain”: effects of cognitive-behavioral therapy on the neural correlates of spider phobia. Neuroimage (2003) 18(2):401–9. 10.1016/S1053-8119(02)00030-7 12595193

[165] Rury V Birchwood M Cochrane R Cognitive therapy and recovery from acute psychosis: a controlled trial: 3. Br J Psychiatry (2000) 177(1):8–14. 10.1192/bjp.177.1.8 10945081

[166] Tarrier N Wykes T Is there evidence that cognitive behaviour therapy is an effective treatment for schizophrenia? Behav Res Ther (2004) 42(12):1377–401. 10.1016/j.brat.2004.06.020 15500811

[167] Jyrä K Knekt P Lindfors O The impact of psychotherapy treatments of different length and type on health behaviour during a five-year follow-up. Psychother Res (2017) 27(4):397–409. 10.1080/10503307.2015.1112928 26829646

[168] Knekt P Lindfors O Härkänen T Välikoski M Virtala E Laaksonen M Randomized trial on the effectiveness of long-and short-term psychodynamic psychotherapy and solution-focused therapy on psychiatric symptoms during a 3-year follow-up. Psychol Med (2008) 38(5):689–703. 10.1017/S003329170700164X 18005493

[169] Psychosis N Schizophrenia in Adults: the NICE guideline on treatment and management, updated edition. London: NICE (2014).

[170] Morrison AP Pyle M Gumley A Schwannauer M Turkington D MacLennan G Cognitive behavioural therapy in clozapine-resistant schizophrenia (FOCUS): an assessor-blinded, randomised controlled trial. Lancet Psychiatry (2018) 5(8):633–64. 10.1016/S2215-0366(18)30184-6 PMC6063993 30001930

[171] Morrison AP Pyle M Chapman N French P Parker SK Wells A Metacognitive therapy in people with a schizophrenia spectrum diagnosis and medication resistant symptoms: a feasibility study. J Behav Ther Exp Psychiatry (2014) 45(2):280–4. 10.1016/j.jbtep.2013.11.003 24440585

[172] Penn DL Meyer PS Evans E Wirth RJ Cai K Burchinal M A randomized controlled trial of group cognitive-behavioral therapy vs. Schizophr Res (2009) 109(1–3):52–9. 10.1016/j.schres.2008.12.009 19176275

[173] Zimmermann G Favrod J Trieu VH Pomini V The effect of cognitive behavioral treatment on the positive symptoms of schizophrenia spectrum disorders: a meta-analysis. Schizophr Res (2005) 77(1):1–9. 10.1016/j.schres.2005.02.018 16005380

[174] Hutton P Cognitive-behavioural therapy for schizophrenia: a critical commentary on the Newton-Howes and Wood meta-analysis. Psychol Psychother (2013) 86(2):139–45. 10.1111/papt.12009 23674465

[175] Newton-Howes G Wood R Cognitive behavioural therapy and the psychopathology of schizophrenia: systematic review and meta-analysis. Psychol Psychother (2013) 86(2):127–38. 10.1111/j.2044-8341.2011.02048.x 23674464

[176] Crawford MJ Killaspy H Barnes TR Barrett B Byford S Clayton K Group art therapy as an adjunctive treatment for people with schizophrenia: a randomised controlled trial (MATISSE). Health Technol Assess (2012) 16(8):iii–iv, 1–76. 10.3310/hta16080 22364962

[177] Lynch D Laws KR McKenna PJ Cognitive behavioural therapy for major psychiatric disorder: does it really work? A meta-analytical review of well-controlled trials. Psychol Med (2010) 40(1):9–24. 10.1017/S003329170900590X 19476688

[178] Gold C Solli HP Krüger V Lie SA Dose-response relationship in music therapy for people with serious mental disorders: systematic review and meta-analysis. Clin Psychol Rev (2009) 29(3):193–207. 10.1016/j.cpr.2009.01.001 19269725

[179] Garety PA Fowler DG Freeman D Bebbington P Dunn G Kuipers E Cognitive--behavioural therapy and family intervention for relapse prevention and symptom reduction in psychosis: randomised controlled trial. Br J Psychiatry (2008) 192(6):412–23. 10.1192/bjp.bp.107.043570 18515890

[180] Talwar N Crawford MJ Maratos A Nur U McDermott O Procter S Music therapy for in-patients with schizophrenia: exploratory randomised controlled trial. Br J Psychiatry (2006) 189:405–9. 10.1192/bjp.bp.105.015073 17077429

[181] Bechdolf A Knost B Kuntermann C Schiller S Klosterkötter J Hambrecht M A randomized comparison of group cognitive-behavioural therapy and group psychoeducation in patients with schizophrenia. Acta Psychiatr Scand (2004) 110(1):21–8 *Erratum in: Acta Psychiatr Scand* (2004) 110(6)483. 10.1111/j.1600-0447.2004.00300.x 15180776

[182] Shahar G Blatt S Ford RQ Mixed anaclitic-introjective psychopathology in treatment-resistant inpatients undergoing psychoanalytic psychotherapy. Psychoanalytic Psychology (2003) 20(1):84–102. 10.1037/0736-9735.20.1.84

[183] Haddock G Barrowclough C Tarrier N Moring J O’Brien R Schofield N Cognitive-behavioural therapy and motivational intervention for schizophrenia and substance misuse. Br J Psychiatry (2003) 183(5):418–26. 10.1192/bjp.183.5.418 14594917

[184] Turkington D Kingdon D, Turner T; Insight into Schizophrenia Research Group. Effectiveness of a brief cognitive-behavioural therapy intervention in the treatment of schizophrenia. Br J Psychiatry (2002) 180(6):523–7. 10.1192/bjp.180.6.523 12042231

[185] Drury V Birchwood M Cochrane R Cognitive therapy and recovery from acute psychosis: a controlled trial. 3. Br J Psychiatry (2000) 177(1):8–14. 10.1192/bjp.177.1.8 10945081

[186] Hogarty GE Greenwald D Ulrich RF Kornblith SJ DiBarry AL Cooley S Three-year trials of personal therapy among schizophrenic patients living with or independent of family, II: Effects on adjustment of patients. Am J Psychiatry (1997) 154(11):1514–24. 10.1176/ajp.154.11.1514 9356558

[187] Buchkremer G Klingberg S Holle R Schulze Mönking H Hornung WP Psychoeducational psychotherapy for schizophrenic patients and their key relatives or care-givers: results of a 2-year follow-up. Acta Psychiatr Scand (1997) 96(6):483–91. 10.1111/j.1600-0447.1997.tb09951.x 9421346

